# Resolving the phylogeny of *Thladiantha* (Cucurbitaceae) with three different target capture pipelines

**DOI:** 10.1186/s12862-023-02185-z

**Published:** 2023-12-12

**Authors:** Mustafa Raza, Edgardo M. Ortiz, Lea Schwung, Gentaro Shigita, Hanno Schaefer

**Affiliations:** Plant Biodiversity Research, Dept. Life Science Systems, Technical University of Munich (TUM), Emil-Ramann-Str. 2, D-85354 Freising, Germany

**Keywords:** Captus, Coalescent, Concatenation, Cucurbitaceae, HybPiper, SECAPR, Target capture, *Thladiantha*

## Abstract

**Background:**

Despite recent advances, reliable tools to simultaneously handle different types of sequencing data (e.g., target capture, genome skimming) for phylogenomics are still scarce. Here, we evaluate the performance of the recently developed pipeline Captus in comparison with the well-known target capture pipelines HybPiper and SECAPR. As test data, we analyzed newly generated sequences for the genus *Thladiantha* (Cucurbitaceae) for which no well-resolved phylogeny estimate has been available so far, as well as simulated reads derived from the genome of *Arabidopsis thaliana.*

**Results:**

Our pipeline comparisons are based on (1) the time needed for data assembly and locus extraction, (2) locus recovery per sample, (3) the number of informative sites in nucleotide alignments, and (4) the topology of the nuclear and plastid phylogenies. Additionally, the simulated reads derived from the genome of *Arabidopsis thaliana* were used to evaluate the accuracy and completeness of the recovered loci. In terms of computation time, locus recovery per sample, and informative sites, Captus outperforms HybPiper and SECAPR. The resulting topologies of Captus and SECAPR are identical for coalescent trees but differ when trees are inferred from concatenated alignments. The HybPiper phylogeny is similar to Captus in both methods. The nuclear genes recover a deep split of *Thladiantha* in two clades, but this is not supported by the plastid data.

**Conclusions:**

Captus is the best choice among the three pipelines in terms of computation time and locus recovery. Even though there is no significant topological difference between the *Thladiantha* species trees produced by the three pipelines, Captus yields a higher number of gene trees in agreement with the topology of the species tree (i.e., fewer genes in conflict with the species tree topology).

**Supplementary Information:**

The online version contains supplementary material available at 10.1186/s12862-023-02185-z.

## Background

Recent advances in phylogenomics enable us to focus on specifically targeted regions of interest in the genome using target capture sequencing [1, 2]. Such an approach is cost-effective as it allows for broad taxon sampling but also has the added benefit of shorter computational time in bioinformatic analyses while increasing sequence coverage [3]. The basic target capture-based method relies on a complex series of interrelated steps of data processing, where baits or probes are designed to hybridize by sequence complementarity with target loci that are sufficiently conserved across the organisms of interest [3–5]. Sequencing of the selected target-specific regions takes place only after the hybridization is complete, and the remaining parts of the genome are discarded [4]. In this context, taxon-specific probes offer higher enrichment success and thus are essential for phylogenetic studies of varying evolutionary depth to increase the accuracy of inferred phylogenetic trees [6]. Moreover, such taxon-specific markers provide a better gene assembly as well as a higher number of segregating sites with the advantage of higher resolution for the identification of gene tree incongruence [6, 7].

Two pipelines, HybPiper [8] and Sequence Capture Processor (SECAPR) [9], have been specifically developed for target capture approaches. HybPiper is currently the most widely used pipeline which can handle target capture data, alone or in combination with genome skimming data (i.e., Hyb-Seq) [10]. A recently developed pipeline, Captus (https://github.com/edgardomortiz/Captus) [11], can handle not only target capture, genome skimming, and Hyb-Seq data, but also RNA-Seq and high-depth whole genome sequencing (WGS) data.

The three pipelines differ in their general strategy: HybPiper starts by matching sequencing reads to target sequences, using BLASTx [12] or DIAMOND [13] if the target is a protein. If the target is a nucleotide sequence, HybPiper uses the alignment algorithm BWA [14]. The groups of reads matching each target sequence are assembled *de novo* independently using SPAdes [15]. In contrast, SECAPR [9] and Captus [11] assemble all reads together using SPAdes [15] and MEGAHIT [16] respectively, and then proceed to find the target loci within the assemblies. SECAPR is limited to matching nucleotide target sequences using BLASTx. In the next step, SECAPR extracts the contigs that match the targets of interest and performs multiple sequence alignments of such contigs across samples using MAFFT [17]. Captus uses Scipio [18] to match protein target sequences or BLAT [19] to match nucleotide target sequences to the assembled contigs. Captus then aligns the extracted markers using either MAFFT or MUSCLE5 [17, 20]. Note that while HybPiper and Captus are designed to deal with amino acid and nucleotide reference targets, SECAPR can only handle nucleotide reference targets containing individual exons (Table 1) [9]. Among the three pipelines, Captus is the only that uses Python’s native parallelization capabilities to process multiple samples simultaneously.

**Table 1:** Major steps of the three pipelines HybPiper, SECAPR, and Captus (differences highlighted in bold)

	HybPiper	SECAPR	Captus
Input	Target Capture (short reads)	Target Capture (short reads)	Target Capture, WGS, RNASeq, Genome Skimming (short reads)
Quality control	**No**	**Yes**	**Yes**
Input target reference	protein or DNA	**only DNA**	protein or DNA
Assembly	De-novo (SPAdes) Separate reads using best locus assembled with SPAdes	De-novo (SPAdes, ABySS) All reads assembled using SPAdes	De-novo **(MEGAHIT)** **All reads assembled using MEGAHIT**
Exon/Intron recovery	**Exonerate**	**BLASTn**	**Scipio**
Paralogs Filter	Yes	Yes	Yes
Alignment	**No**	**Yes**	**Yes**

The three pipelines were used to evaluate phylogenetic relationships using coalescent and concatenation methods. In a genus-level study like ours, it is important to contrast both methods because the multispecies coalescent is expected to perform better in the presence of incomplete lineage sorting (ILS) and hybridization events [21]. The coalescent, however, can be affected by a high degree of gene conservation which results in alignments with limited phylogenetic signal and consequently, poorly resolved gene trees. In such cases, concatenation is expected to minimize the stochastic variation among genes trees with the drawback of assuming all genes evolved under the same history, and therefore disregarding ILS or hybridization [22–26]. The concatenation and coalescent tree topologies can be used to compare the performance of different pipelines, which, in theory, should produce congruent tree topologies.

Here, we chose the genus *Thladiantha* from the tribe Thladiantheae in the family Cucurbitaceae as the test group [27]. *Thladiantha* is native to temperate and tropical Asia and includes 27 accepted species (https://powo.science.kew.org/taxon/urn:lsid:ipni.org:names:13424-1). Previous phylogenetic studies included 25 *Thladiantha* species but only few DNA regions [27] or full transcriptomes of five species [28]. All studies confirmed *Thladiantha* as a monophyletic group [27, 28] and revealed a sister group relationship with *Baijiania* while both genera are then sister to *Indofevillea*. Our aim was to produce a new target capture and genome skimming dataset for *Thladiantha* and analyze it with Captus, HybPiper, and SECAPR to compare the performance of the three pipelines and, at the same time resolve the phylogeny of *Thladiantha*.

Since the reference target sequences used by these pipelines are usually from distant relatives, the accuracy of the recovered gene structures cannot be properly determined. We therefore performed an additional test in which we simulate sequencing reads from the *Arabidopsis thaliana* genome and compare the genes obtained from the three pipelines with the published gene annotation for *Arabidopsis*.

We compare (1) the number of informative sites in nucleotide alignments, (2) the time needed for assembly and extraction, (3) the locus recovery rate, and (4) the topologies of the nuclear and plastid phylogenies based on concatenation and coalescent methods. Finally, we performed morphological analyses of character evolution in *Thladiantha* on our preferred phylogeny estimate.

## Results

### Summary statistics of the three pipelines

The total number of genes recovered from the 30 *Thladiantha* samples (target capture) with Captus ranged from 597 to 1176, and from 520 to 1168 for SECAPR. The total number of genes recovered with HybPiper BLASTx and HybPiper DIAMOND ranged from 468 to 1150. The average proportion of the total gene length recovered with Captus was 87%, and with SECAPR it was 76%. The average proportion of the total gene length recovered with HybPiper BLASTx and HybPiper DIAMOND was 86%. The number of genes recovered in each sample was higher in Captus than in SECAPR, HybPiper-BLASTx and HybPiper-DIAMOND (Table 2, Additional file 1: Table S4).

**Table 2:** Recovery statistics for HybPiper, SECAPR, and Captus

Pipeline	No. of genes	Average length of alignments	No. of nucleotides	No. of informative sites
HybPiper-BLASTx	1,178	2,487	2,929,586	216,707
HybPiper-DIAMOND	1,178	2,483	2,925,302	216,213
SECAPR	1,180	1,543	1,821,303	241,889
Captus	1,180	2,883	3,402,068	300,122

### Comparison of the three pipelines

#### Speed

**Fig. 1:**
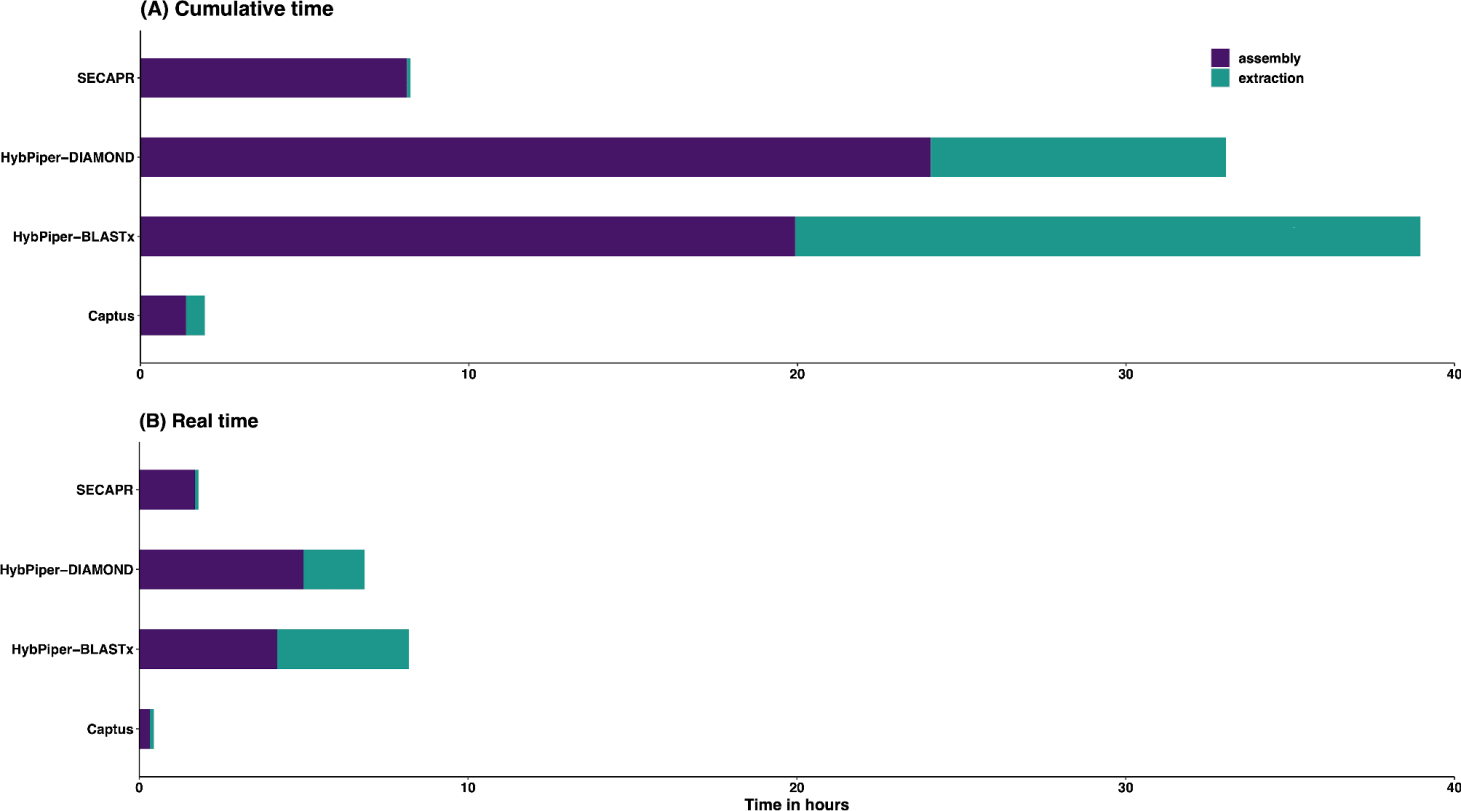
Computation speed in **(A)** cumulative time (sum of the time taken for each sample independently) and **(B)** real-time (the wall clock time from start to finish of the process) for the different pipelines

We used our *Thladiantha* data, and the simulated *Arabidopsis* reads to evaluate the speed of all three pipelines for the assembly of reads and extraction of gene regions in real (the wall clock time from start to finish of the process) and cumulative time (sum of the time taken for each sample independently). In cumulative time with the *Thladiantha* data, SECAPR was 5.4 times faster than Captus and 179 times faster than HybPiper-BLASTx for the extraction. HybPiper’s assembly time was longer than in the other two pipelines (Fig. 1A; Additional file 1: Table S2). HybPiper took the longest time for extraction and for assembly. In parallel processing, Captus was c. 18.5 times faster in real-time than HybPiper-BLASTx, 15.5 times faster than HybPiper-DIAMOND, and 4.06 times faster than SECAPR (Fig. 1B). In cumulative time, considering assembly and extraction steps together, Captus was 19.9 times faster than HybPiper-BLASTx, 16.9 times faster than HybPiper-DIAMOND, and 4.19 times faster than SECAPR.

In terms of assembly and extraction times with the simulated *Arabidopsis* dataset, Captus was c. 8 times faster than HybPiper-BLASTx, 3.9 times faster than HybPiper-DIAMOND, and 4 times faster than SECAPR in cumulative time. HybPiper with BLASTx took the longest time for extraction and assembly among the three pipelines (Additional file 1: Table S3).

#### Locus recovery

**Fig. 2:**
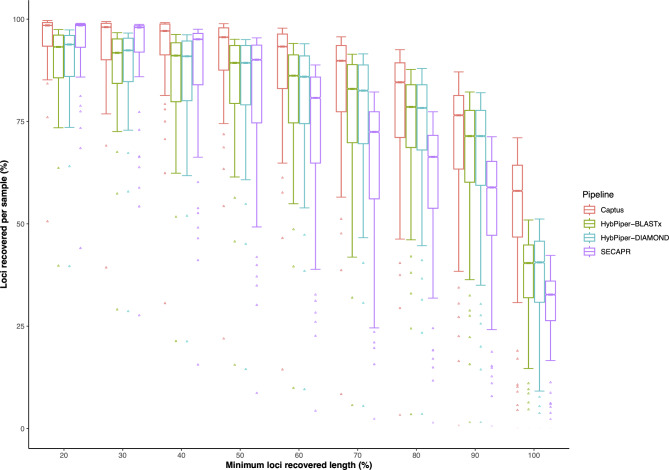
Locus recovery for the different pipelines. Recovered loci are sorted in bins ranging from 20–100% recovered length, as the minimum coverage threshold tested was 20

For the *Thladiantha* data, locus recovery decreases as we increase the minimum locus coverage threshold (Fig. 2). The overall performances of HybPiper with BLASTx and DIAMOND were comparable with c. 86–94% of the loci recovered for thresholds up to 60% locus length. The locus recovery then decreased to 40% for the full locus length for HybPiper (Fig. 2). Up to a minimum of 50% recovered length per locus, SECAPR recovered 90–98% of the loci for *Thladianta*. The performance of SECAPR then drastically decreased for locus length > 50%, and it recovered only 33% of the loci at full length (Fig. 2). The total loci retained by Captus ranged between 84 and 98% for up to a minimum of 80% recovered length (Fig. 2). The locus recovery reached 58% at full-length for Captus.

**Fig. 3:**
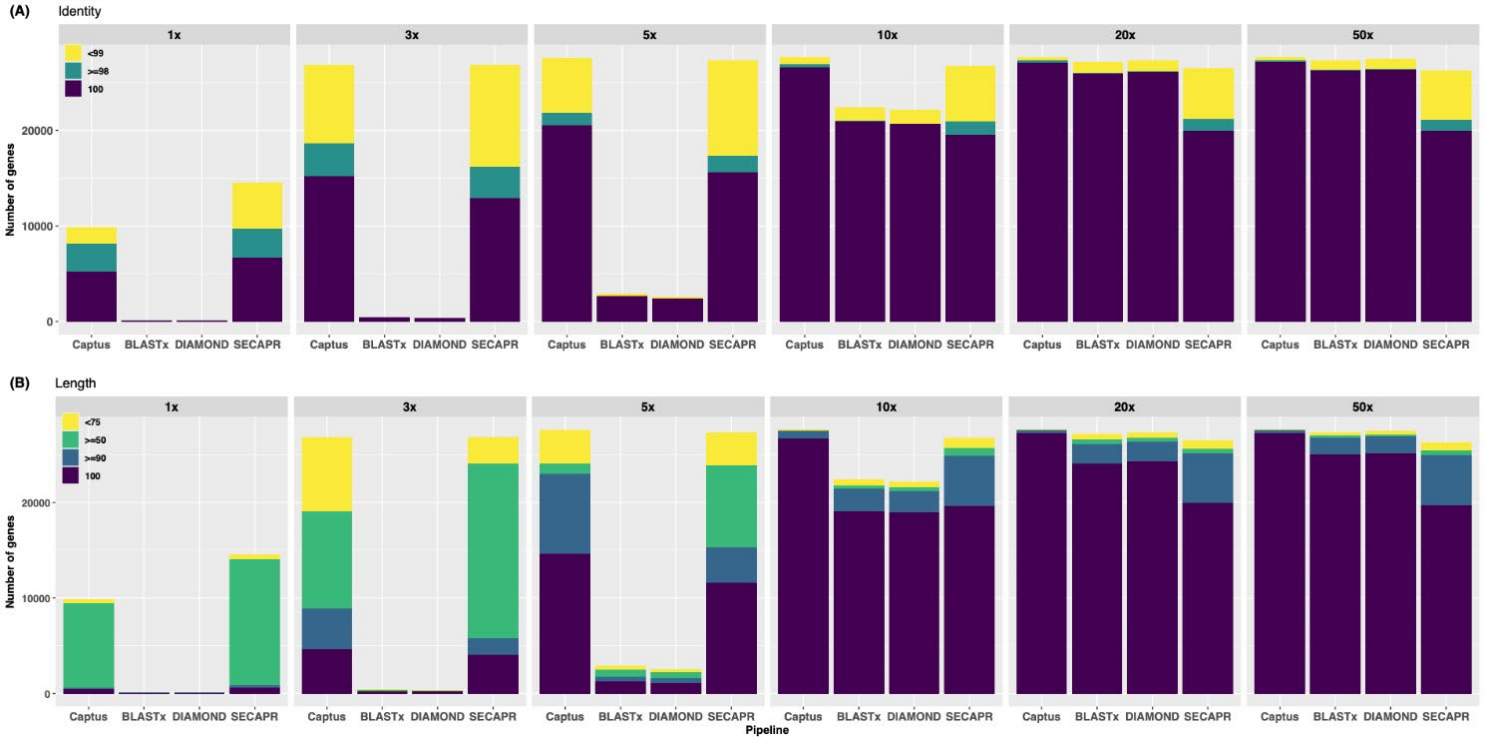
Performance of the different pipelines in **(A)** percent sequence identity **(B)** sequence length at different sequencing depths (1x, 3x, 5x, 10x, 20x, 50x) using simulated *Arabidopsis* reads

With the *Arabidopsis* data, locus recovery was equally high for all three pipelines at 20x and 50x sequencing depths. SECAPR and Captus recovered many more loci than HybPiper at very low depths (Fig. 3, Additional file 1: Figure S1). When the sequencing depth was increased from 10x to 50x, the recovery drastically increased for HybPiper-BLASTx and HybPiper-DIAMOND (Fig. 3, Additional file 1: Figure S1). We then evaluated the performance of pipelines in terms of sequence length and identity across different depths from 1x to 50x for *Arabidopsis thaliana* (Fig. 3). The overall performances of pipelines for *Arabidopsis* across different depths follow a similar trend as in Fig. 2. Except for very low depth (1x), Captus consistently recovered near-perfect sequence accuracy, length, and completeness at depths ranging from 3x to 50x (Fig. 3). Using the --cov_cutoff 4 option, as suggested by the authors of HybPiper [8] we find that locus recovery increased in HybPiper BLASTx and HybPiper DIAMOND at 10x coverage, but at the cost of decreased accuracy (Additional file 1: Figure S18). Lowering the coverage cutoff below 4 did not increase the recovery.

#### Alignments and number of informative sites

For the *Thladiantha* ingroup, we obtained a total of 117,397,818 clean reads from target capture and 200,964,766 clean reads from genome skimming. Using our curated *Thladiantha*-specific references containing 1180 loci, Captus and SECAPR recovered all 1180 gene regions, while HybPiper missed two of them. Alignment lengths and the number of nucleotides were similar in HybPiper-BLASTx and HybPiper-DIAMOND, whereas SECAPR alignments were on average only half as long. The number of parsimony informative sites is the highest in the alignments from Captus (Table 2, Additional file 1: Table S4).

## Phylogenetic tree estimation for ***Thladiantha*** with three pipelines

### Nuclear phylogeny

#### Phylogenies from concatenated alignments

**Fig. 4:**
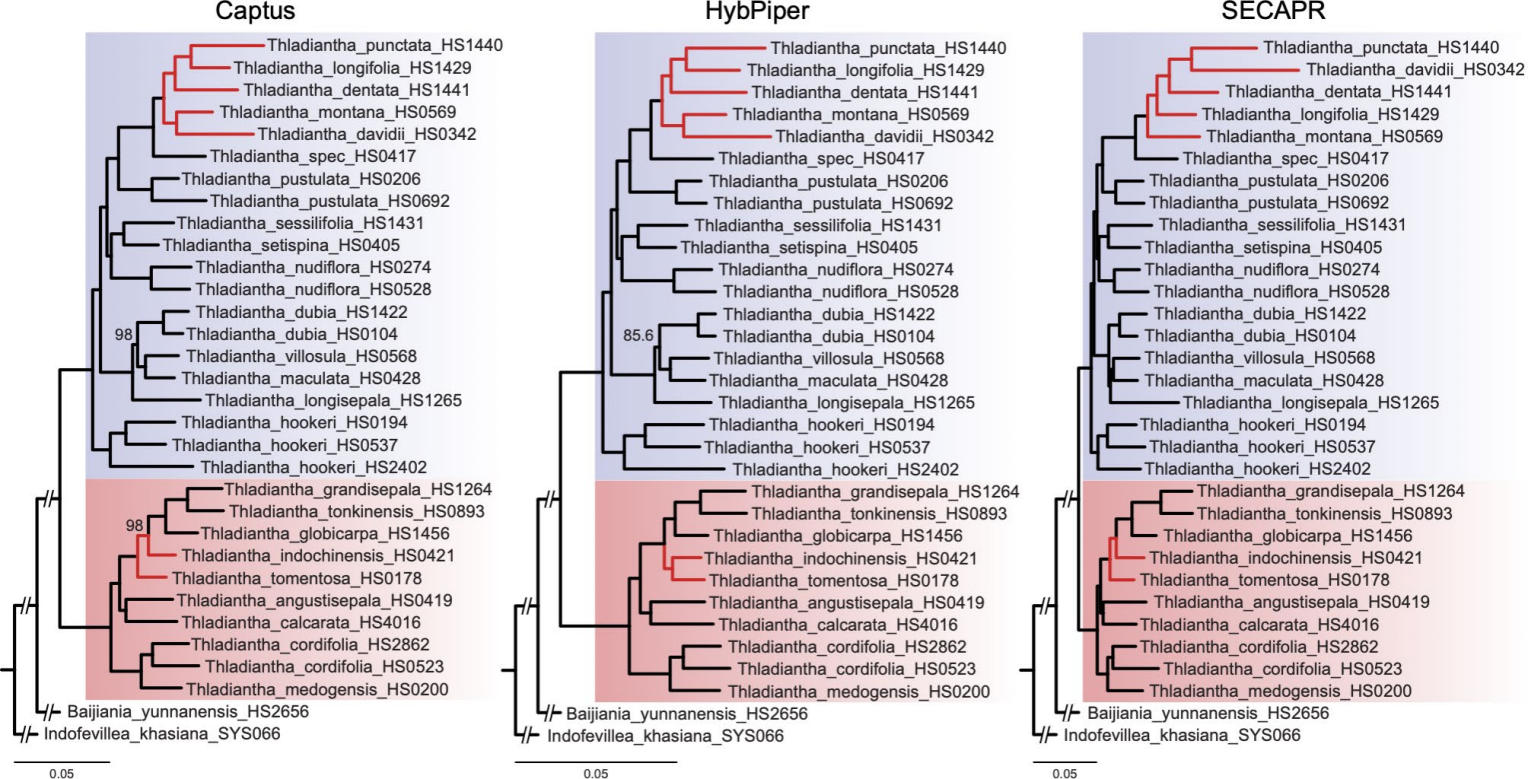
Concatenated nuclear phylogenies of *Thladiantha* based on the Captus, HybPiper, and SECAPR alignment. Group 1 (section *Thladiantha*) highlighted in blue, group 2 (section *Fidobractea*) in red, bootstrap values below 100 indicated at the nodes. Topological conflict between the pipelines indicated by red branch colour

The maximum likelihood (ML) analysis of the concatenated nuclear alignments yielded a well-resolved phylogeny for the datasets from all three pipelines, with all *Thladiantha* samples grouped into two clades (Fig. 4, group 1 highlighted in blue, group 2 highlighted in red). Most of the phylogenetic relationships between species are strongly supported [i.e., ultrafast bootstrap (UFBS) values of 100] in all phylogenies (Fig. 4). However, each of the pipelines produced a slightly different tree topology. The tree topologies obtained with HybPiper and Captus differ in group 2, where *T. tomentosa* is sister to *T. indochinensis* in the HybPiper tree, while in the Captus tree it is sister to a clade comprising *T. indochinensis, T. globicarpa, T. tonkinensis*, and *T. grandisepala* (Fig. 4). In the SECAPR tree, the topology of group 2 is identical to Captus, while in group 1, the taxonomic positions of six species (*T. punctata, T. davidii, T.* spec. HS0417, *T. dentata, T. longifolia*, and *T. montana*) differ from the HybPiper and Captus topologies (Fig. 4).

#### Multispecies coalescent (MSC) phylogenies

**Fig. 5:**
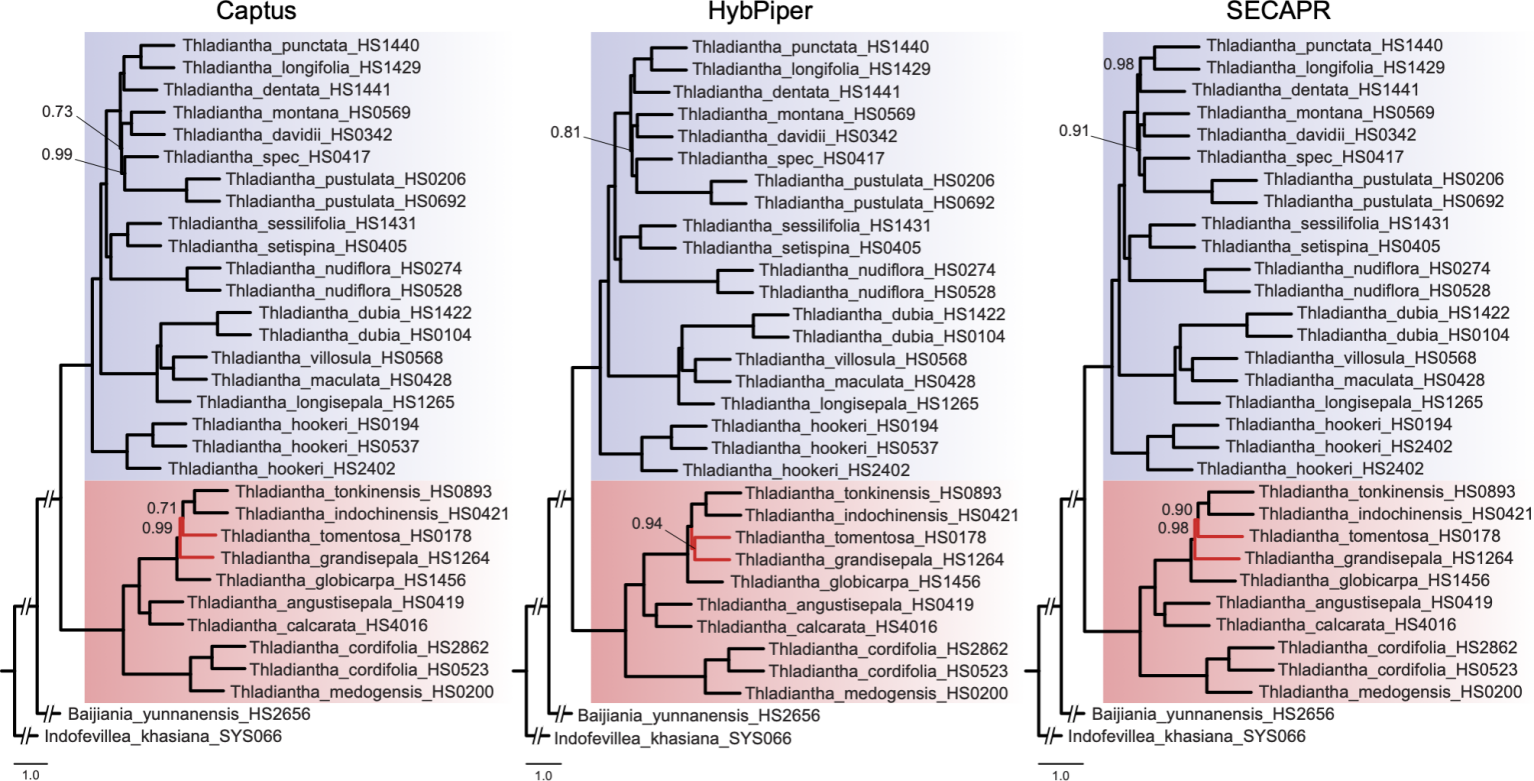
Coalescent nuclear phylogeny of *Thladiantha* based on the Captus, HybPiper, and SECAPR alignment. Group 1 (section *Thladiantha*) highlighted in blue, group 2 (section *Fidobractea*) in red, posterior probabilities below 1 indicated at the nodes. Topological conflict between the pipelines indicated by red branch colour

Using the MSC based approach, the nuclear alignments also yielded well-resolved species trees with all three pipelines. Again, all *Thladiantha* samples grouped into the two clades found in the concatenated analysis (Fig. 5). Most of the phylogenetic relationships between species are strongly supported in all three trees (Fig. 5). The tree topologies obtained with HybPiper, Captus, and SECAPR are identical for group 1, although branch lengths and node support differ slightly (Fig. 5). For group 2, the HybPiper tree differs from the SECAPR and Captus trees in the taxonomic placements of three species (*T. tomentosa*, *T. grandisepala*, and *T. globicarpa*). In the HybPiper tree, *T. tomentosa* is found to be sister to *T. grandisepala* with local posterior probability (LPP) of 0.77, while in Captus and in SECAPR trees, *T. grandisepala* is sister to the clade comprising *T. tomentosa*, *T. tonkinensis*, and *T. indochinensis* with LPP of 0.98 and 0.99, respectively.

#### Comparison between concatenation and coalescent phylogenies

Comparing all six nuclear phylogenies from the three pipelines (Figs. 4 and 5) based on the concatenation and coalescent approaches, we found that for group 1, the phylogenetic positions of 13 *Thladianta* species are in congruence across all the six phylogenies, while they are incongruent for *T. punctata, T. davidii, T.* spec. HS0417, *T. dentata, T. longifolia, T. pustula*, and *T. montana*. In group 2, the positions of *T. medogensis*, *T. cordifolia*, *T. angustisepala*, and *T. calcarata* are in agreement across all the six phylogenies.

#### Plastid phylogeny

Plastid phylogenies could only be inferred using the pipelines HybPiper and Captus since SECAPR cannot extract unique contigs for each locus during the extraction process. The two obtained plastid phylogeny topologies (Additional file 1: Figure S2) largely differ from the inferred nuclear trees (Figs. 4 and 5): the split in two groups is not supported in the plastid phylogenies since *T. medogensis* is found to be sister to all other *Thladianta* species in both the HybPiper and Captus trees with UFBS = 100 (Additional file 1: Figure S2). In the HybPiper plastid tree, most of the phylogenetic relationships between species are poorly supported, with a UFBS range of 19 to 67. In the Captus tree, the relationships within group 1 are also poorly supported with a UFBS range from 40 to 61 (Additional file 1: Figure S2). Among the ten *Thladianta* species of group 2, the Captus plastid phylogeny supports the grouping of *T. indochinensis*, *T. tonkinensis*, *T. globicarpa*, and *T. tomentosa*, *T. grandisepala*, *T. calcarata*, and *T. angustisepala*, although the topologies differ (Figs. 4 and 5). The plastid phylogenies and nuclear phylogenies show that the *T. cordifolia* clade is strongly supported, but in the Captus and HybPiper plastid phylogeny, the placement of this clade has a low UFBS support of 51 and 35 (Figs. 4 and 5; Additional file 1: Figure S2). *Thladiantha indochinensis* and *T. tonkinensis* are sister species in the plastid tree and in the coalescent-based nuclear phylogenies of SECAPR, Captus, and HybPiper (Fig. 5). The relationship between *T. pustulata* and *T.* spec. HS0417 in the plastid phylogenies is also found in the coalescent-based nuclear phylogenies. *Thladiantha villosula* and *T. maculata* are found to be sister species in the plastid phylogenies (UFBS = 100 in HybPiper and UFBS = 91 in Captus) as well as in the coalescent-based nuclear phylogenies (Fig. 5).

#### Gene tree conflict and phylogenetic signal

**Fig. 6:**
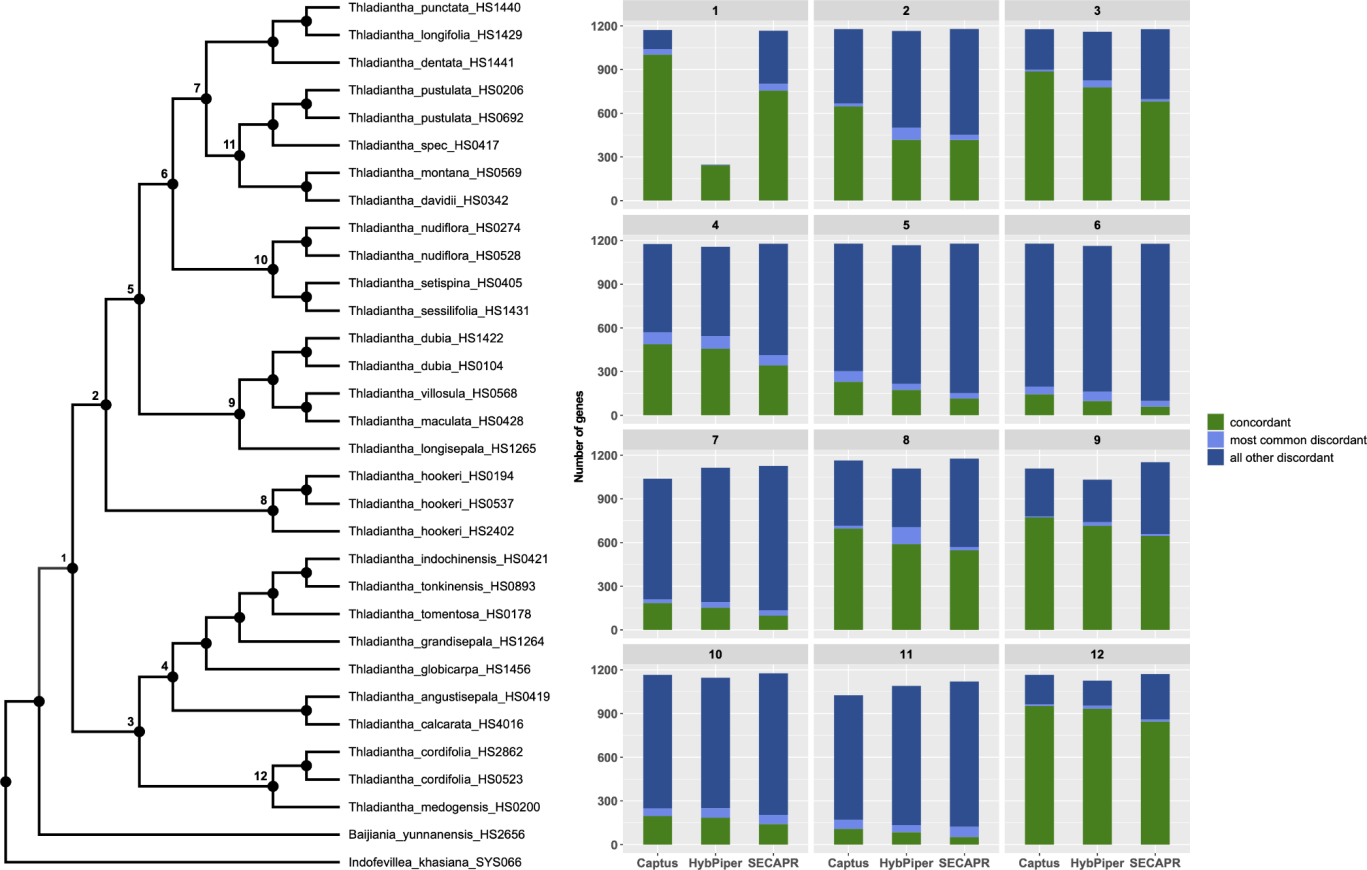
Nuclear species phylogeny (left) and summarized conflict analysis (right) using PhyParts. The bar graph represents the concordant and discordant genes of the numbered nodes for each of the three pipelines

The conflict analysis of the nuclear dataset revealed that 1003 out of 1171 (85.7%) informative gene trees support the monophyly of *Thladiantha* in Captus, 756 out of 1167 (64.8%) in SECAPR, and 246 out of 247 (99.6%) in HybPiper (node 1 in Fig. 6). The number of informative gene trees for the monophyly of *Thladiantha* was much lower in HybPiper than in the other two pipelines due to the lower locus recovery of the outgroups (node 1 in Fig. 6). The tree topologies inferred from the alignments of the three pipelines all show the deep split into two clades (nodes 2 and 3 in Fig. 6): in Captus, group 1 with a concordance value of 647 out of 1178 (54.9%) and group 2 with a value of 887 out of 1177 gene trees (75.3%) (Fig. 6). Within the two clades, most of the gene trees agree with the clade topology of the species tree. In SECAPR, group 1 with a concordance value of 416 out of 1179 (35.2%) and group 2 with a value of 681 out of 1177 gene trees (57.8%) (Fig. 6). In HybPiper, group 1 with a concordance value of 420 out of 1165 (35.9%) and group 2 with a value of 770 out of 1160 gene trees (66.3%) (Fig. 6).

#### Phylogenetic network

The SplitsTree network derived from the concatenated nuclear supermatrix confirmed the two *Thladiantha* groups in Captus, HybPiper, and SECAPR but also revealed 98, 99, and 86 conflicting splits, respectively (Additional file 1: Figures S3, S4, S5). In group 1, a split in four clades with basal reticulation is inferred, whereas group 2 is composed of two clades with reticulation events.

#### Ancestral character states

We traced the ancestral states of 12 morphological characters (Additional file 1: Table S5) under the parsimony model in Mesquite v3.70 [47] using the alignment of 1180 concatenated nuclear loci. Leaf blade shape is inferred as ovate-cordate in the ancestor, with transitions in *T. cordifolia*, *T. sessilifolia*, and *T. longifolia*. Male and female calyx segments were most likely lanceolate in the ancestral state. The ancestral shape of male corolla segments is ovate, with one independent transition to an oblong shape. An oblong anther was inferred to be the ancestral state in the genus with transitions to elliptic anthers in the clade *T. sessilifolia, T. setispina*, and in *T. dentata, T. davidii*, and *T. globicarpa.* Oblong fruit shape is inferred to be the ancestral state, with independent evolution of the ovoid state in three species (Additional file 1: Figures S6-S17).

## Discussion

### Speed

Based on both the real- and cumulative-time analyses, we found that Captus outperformed the other two pipelines for assembly and extraction, followed by SECAPR and HybPiper. The simulated *Arabidopsis thaliana* genome reads confirmed this trend. SECAPR is the fastest pipeline for extraction of gene regions. Captus, however, is faster in assembly of reads (Fig. 1; Additional file 1: Tables S2, S3). This is possibly because Captus uses python parallelization libraries to process and analyze multiple samples simultaneously. The choice of assembler is significant too. MEGAHIT normally outperforms other assemblers in speed [29]. Natively, Captus can handle the tasks following user-specified arguments of threads and parallelization, which is not the case for HybPiper and SECAPR.

### Locus recovery

For the *Thladiantha* dataset, Captus consistently recovered greater numbers of loci per sample than HybPiper or SECAPR (Fig. 2). This can possibly be attributed to the use of MEGAHIT which is fast, but also allows that a larger portion of the genome to be recovered completely [29]. One of the main reasons for an improved recovery for Captus could be that MEGAHIT allows *de novo* assembly of all reads together (instead of just assembling groups of reads that match the coding parts of the reference sequences) in a precise and timely manner. Moreover, the use of Scipio in Captus enables it to efficiently handle fragmented assemblies from the target capture data, unlike Exonerate in HybPiper and BLASTn in SECAPR (Table 1) [12, 18, 30]. Scipio determines the precise gene structure from a protein sequence and a genome sequence based on the alignments from BLAT. Even when sequencing errors or incomplete genome assemblies lead to hits that stretch across multiple contigs, Scipio provides improved prediction accuracy compared to BLAT and Exonerate and gives the user an accurate determination of intron-exon borders and splice sites, even correcting for shifts in reading frame. In *Arabidopsis thaliana*, our results show that SECAPR and Captus are more sensitive throughout depth thresholds, though Captus consistently recovered 97.21–99.94% of genes among all the pipelines for a wide range (3x to 50x) of sequencing depths (Additional file 1: Figure S1). Also, Captus consistently recovered the perfect sequence quality length and sequence identity across different depths from 3x to 50x (Fig. 3).

While SECAPR and Captus recovered equal numbers of genes for our test data, the average length of alignments was shorter in SECAPR than that of the Captus alignments because SECAPR and Captus used different tools to trim the alignment of contigs in the alignment step. In consequence, the number of informative sites was higher for Captus than for SECAPR or HybPiper.

### Tree topology

Regarding the resulting topologies, the results are less clear, which could be due to biological reasons and not methodological problems. *Thladiantha* was found to be monophyletic like in all the previous studies [27, 28]. The deep split in two clades matches the morphological classification of the genus which introduced two sections based on the presence or absence of flabelliform male bracts, namely section *Fidobractea* and section *Thladiantha* [31]. Our group 1 represents the morphological section *Thladiantha*, while our group 2 matches section *Fidobractea*. The species in section *Thladiantha* are restricted to China, with an altitude range from 300 to 3500 m, while the species in section *Fidobractea* are distributed from southern China to east India, Laos, Myanmar, Thailand, Sumatra and Java, with an altitude range of 800–2600 m. The topology inferred by Guo et al. [28] based on full transcriptome of five species is in agreement with our nuclear phylogenies, while an earlier comprehensive phylogenetic study did not find the deep split of *Thladiantha* into two clades, most likely due to the fact that it used only a few DNA regions mainly from the plastome [27].

Phylogenies based on concatenation and coalescent methods yielded incongruent trees. The concatenation approach assumes that all loci share a common evolutionary history which is not the case in the presence of ILS [23, 25]. The coalescent method is able to handle ILS better [21, 25, 26]. Therefore, it is likely that our coalescent trees show a more realistic topology. Captus and SECAPR yielded identical topologies with the coalescent method, whereas the HybPiper alignments resulted in a different topology. These differences in coalescent topologies among pipelines are most probably caused by the different assembly strategies. HybPiper filters reads before assembly: for each sample, reads are mapped against the target genes separately, and only those reads that match the target locus are kept. Matching reads are then assembled into separate directories for each gene. Captus and SECAPR assemble *de novo* all the reads into contigs first and then match the corresponding target sequences.

Regarding plastid phylogenies, we could only compare Captus and HybPiper, since SECAPR produced redundant contigs during the extraction step for each locus, and the resulting alignments were chimeric. Among the two plastid phylogenies, we found better clade-support in the Captus tree than in the HybPiper tree (Additional file 1: Figure S2). Comparing the plastid phylogenies to the coalescent-based nuclear trees (Fig. 5), eight species have congruent relationships in the plastid phylogenies. However, the deep split into two clades found in the coalescent-based nuclear phylogenies is not supported in the plastid phylogenies and most of the *Thladiantha* species are differently placed in the plastid phylogenies (Fig. 5; Additional file 1; Figure S2). This indicates incongruence between the plastid and nuclear evolutionary history in *Thladiantha*.

In the gene trees to species tree conflict analysis, we found internal incongruence in the coalescent trees from all three pipelines (Fig. 6). However, in Captus, we found less incongruence compared to HybPiper and SECAPR. This incongruence is not only due to biological reasons but due to methodological reasons, including the quality of the assembly, which also affects the quality of the phylogenetic trees. The higher nodal congruence in the species tree from Captus is probably due to the better quality of the assembly. Therefore, we speculate that most of the remaining incongruence for Captus is most likely biological and not methodological. These findings imply that even a small number of gene trees can yield a consistent species tree. The biological reason for incongruence in the trees could be hybridization, which can also be seen in our network analysis. The SplitsTree network grouped the species in two groups like in the nuclear phylogenetic trees (Additional file 1: Figures S3, S4, S5) but detected several basal reticulation events. The detected reticulations suggest that our inference of ancestral character states should be interpreted cautiously since the bifurcating tree topology used in the analysis represents an incomplete picture of the evolutionary history of the lineages.

## Conclusions

In terms of speed and overall gene recovery, Captus was found to be the best choice among the three pipelines for both the empirical dataset of *Thladiantha* and the simulated dataset of *Arabidopsis thaliana*. Regarding the number of informative sites in nucleotide alignments, the performance of pipelines decreased from Captus to SECAPR to HybPiper. The comparison of the tree topologies revealed that there are very minor topological differences between the *Thladiantha* species trees produced by the three pipelines. However, Captus produced a higher number of gene trees that agree with the species tree nodes. The preferred MSC-based nuclear species tree revealed consistent relationships between 28 *Thladiantha* species among all three pipelines, except for the conflicting placements of *T. tomentosa* and *T. grandisepala*. While the nuclear tree topology is well-resolved, most of the species are unplaced in the plastid trees.

More generally, the simple installation and operation process of Captus, with speed optimized at every step, allows users to analyze either raw data or cleaned/assembled reads and add samples to existing datasets. HybPiper, on the other hand, requires cleaned reads in FASTQ format and several hours or days to assemble and extract. Subcommands are needed for downstream analysis and external tools for alignment. SECAPR provides detailed logs and reports, but it takes longer to generate reports using subcommands. SECAPR can only use nucleotide sequences as a reference with one sequence per locus of interest. We conclude that Captus is the most user-friendly pipeline tested in this study.

## Methods

### Taxon sampling and sequencing

We analyzed a total of 30 herbarium samples of *Thladiantha*, two types of data (target capture and genome skimming), plus one sample each for the outgroups *Baijiania yunnanensis* and *Indofevillea khasiana* based on Schaefer and Renner [27]. For the outgroup, genome skimming data with the SRR10137784 and SRR10137792 from Bellot et al. [32] were used. The specimen information is shown in Table S1. Total genomic DNA was isolated from dry leaf material with NucleoSpin® Plant II kit (MACHEREY-NAGEL, Düren, Germany), following the manufacturer’s protocol. Rapid Genomics LLC (Florida, U.S.A.) performed the library preparation for all 30 samples of target capture and genome skimming data using a probe set designed by EMO.

### Raw data processing

Quality filtering and trimming of the raw reads were performed using the “clean” function of Captus v0.9.83 based on BBduk of BBTools [33] with default settings. To trim with Captus, adapters were first removed in two rounds. The leading and trailing read regions of the adapter-free reads were then trimmed using an average PHRED quality score threshold of 13. After quality trimming, reads with an average PHRED quality score below 16 were removed.

### ***Thladiantha***-specific reference 

Initially, we used target references from the available transcriptomes of Cucurbitaceae, including the species *Citrullus lanatus, Cucumis melo, Cucumis sativus, Cucurbita maxima, Cucurbita moschata, Cucurbita pepo, Lagenaria siceraria, Momordica charantia*, and *Thladiantha villosula*. For the construction of new reference targets, we used the “assemble” function of Captus to assemble paired reads into contigs, DNA regions were extracted using the “extract” function in Captus with --nuc_min_identity 55 and --nuc_min_coverage 20 to match the contigs to the reference targets. The regions were then aligned using the “align” function of Captus and each locus was clustered using MMseqs2 v.13-45111 [34]. We clustered the data using a custom python script that ran in two rounds. We used --min_seq_id 0.895 and --cluster-mode 0 in the first round, and a --min_seq_id 0.95 and --cluster-mode 2 in the second round. We kept only the clusters with at least four samples per locus and discarded the others. However, if the locus had more than one cluster due to the presence of paralogs, we considered them as new loci. Following that, we used --cluster-mode 2 and --cov_mode 1 -c 0.8 to reduce the number of representatives per cluster, and these clusters became the new *Thladiantha*-specific reference loci.

### Assembly, extraction, and alignment

#### Captus

We used the “assemble” function in Captus to assemble paired reads into contigs (*de novo* assembly) by MEGAHIT v1.2.9 [16]. The target genes were extracted using the “extract” function in Captus v0.9.90 with --nuc_min_identity 90 and --nuc_min_coverage 20, and for outgroups used the default settings to match the contigs to *Thladiantha*-specific reference targets using Scipio v1.4 [18]. The “align” function aligned the extracted markers using MAFFT v7.505 [17] and trimmed them with the ClipKIT v1.3.0 with default settings in Captus [35].

#### HybPiper

The raw reads were cleaned with Captus and then analyzed in HybPiper v2.0.1 [8] using BLASTx v2.12.0 [12] and also with DIAMOND v2.0.15 [13], with the respective target sequences as the references. We then assembled those reads using SPAdes v3.15.3 [15] with the “hybpiper assemble” function. Exonerate v2.4.0 [30] was subsequently used to extract the coding sequences from contigs using the specific targets with --thresh 90. For the outgroups with genome skimming data we used the setting --depth_multiplier 0 and --cov_cutoff off. The extracted sequences were aligned using the “auto” option in MAFFT [17] and trimmed with ClipKIT v1.3.0 using the parameters --mode smart-gap [34]. In HybPiper, we also observed a minor difference between the trimmed and untrimmed phylogeny alignments.

#### SECAPR

The raw reads were cleaned with Captus and then assembled into contigs using the “assemble_reads” function in SECAPR v2.2.8 [9] with SPAdes v3.15.2 [15]. The extraction was performed using the “find_target_contigs” function in SECAPR using the nucleotide target loci and mapping the contigs to target sequences using BLASTn with – min_identity 90, and for outgroups used the default settings. The “align_sequences” function aligned the extracted markers using MAFFT [17] and trimmed them using trimAl [36] with default settings in SECAPR.

### Pipeline comparisons

HybPiper, SECAPR, and Captus were run with the target capture dataset of the 30 *Thladiantha* samples as input, using 12 CPUs for each sample, five samples concurrently, and a total of 60 CPUs utilized for the comparison [37]. Captus natively used python parallelization using the concurrent option during the analysis; we ran the HybPiper and SECAPR using the parallel -j (run five jobs in parallel) option in Linux [37]. To obtain text outputs in the same format, we equalized the settings of the different pipelines in R studio v2022.07.0. For this, we used intronerate.gff from HybPiper, selected_blast_hits.txt from SECAPR, and captus-assembly_extract.stats.tsv from Captus. To remove the overlapping hits in the BLASTn results (i.e., subject overlap and query overlap) of SECAPR [9], we calculated the percentage of protein recovery by removing the overlapped regions using a custom python script. We selected the best hit for each locus from each pipeline based on a sequence identity threshold of 90% and a minimum coverage of 20%. To select the best hits, we used the product of identity and protein recovery percentage. We calculated the time for each pipeline using the ‘lubridate’ package of R [38] and analyzed the cumulative (sum of the time taken for each sample independently) and real-time (the wall clock time from start to finish of the process). We used the ggplot2 [39] function in R for visualisation.

#### Efficiency test with ***Arabidopsis thaliana*** data

For a further assessment of the efficiency of the three pipelines, we tested how accurate the gene detection of each pipeline was at different simulated sequencing depths using a known genome and known annotations of *Arabidopsis thaliana*. We compared the accuracy, the number of recovered genes, and the recovered length of the genes using simulated read data from *Arabidopsis thaliana*. We did this by downloading the assembly TAIR10.1 from NCBI (https://www.ncbi.nlm.nih.gov/data-hub/genome/?taxon=3702) and producing simulated reads with bbmap v38.97 [33] using the function randomreads.sh. A total of 39,889,544 paired-end reads with 150 bp length were generated with a general error rate of mapping 0.27%. We subsampled the reads using the bbmap reformat.sh function with different sequencing depths (1x, 3x, 5x, 10x, 20x, and 50x). We downloaded *Arabidopsis thaliana* nucleotide coding sequences and retained the longest coding sequences for each gene, and the resulting 27,620 coding sequences were used as the reference targets. The pipelines HybPiper, SECAPR, and Captus were run on Linux using 64 CPUs with the simulated dataset of the *Arabidopsis thaliana* as input.

### Phylogenetic inference

#### Gene tree estimation

We estimated phylogenetic trees for each individual alignment for the nuclear genes (exons plus introns) with the three different pipelines using ML in IQ-TREE v2.0.6 [40]. We used the Akaike information criterion (AIC) for model selection [41] to select the appropriate nucleotide substitution model during the run with -m TEST. The UFBS replicates with option -bb 1000 were used to evaluate node support [42].

#### Species tree estimation

We ran ML searches to estimate species trees from the concatenated gene alignments, IQ-TREE can automatically concatenate them into a supermatrix prior to analysis by using option -p. Nucleotide models were estimated using each gene as a partition in IQ-TREE [40]. We used the Akaike information criterion (AIC) for model selection [41]. We optimized and sped up the partitioning with 1000 ultrafast bootstrap replicates with the settings: -nt 12 --seed 123 -m TESTMERGE -rcluster 10 -AIC -bb 1000 in the rcluster algorithm.

For the coalescent-based approach, we used ML gene trees calculated separately using IQ-TREE with 1000 ultrafast bootstrap replicates [42]. A species tree was inferred from the gene trees with ASTRAL-Pro v1.1.6 [43] using maximum quartet support for species tree calculation with local posterior probability on nodes.

#### Plastome phylogeny estimation

We used the genome skimming data to infer a third type of phylogeny. We followed the same procedure as described above for the nuclear dataset to assemble and extract the plastome dataset using Captus and HybPiper but used a different sequence identity threshold of 55%. We used the curated target loci SeedPlantsPTD.FNA, which is available in Captus, containing a representative set of chloroplast proteins of seed plants. Plastid regions were extracted using the respective target loci, and extracted regions were aligned with the “align” function using MAFFT [17] and trimmed with ClipKIT v1.3.0 using default settings in Captus [35], and in HybPiper used the untrimmed data. All the nucleotide alignments were analyzed with IQ-TREE [40], which automatically concatenates them into a supermatrix prior to analysis to estimate the species tree.

#### Conflict analysis

To identify gene tree concordance and discordance patterns in the nuclear coalescent phylogeny, we used PhyParts v0.0.1 [44]. PhyParts maps the gene trees on the species tree to identify the number of concordant, conflicting, and uninformative gene trees for each node of the species tree. We mapped the nuclear gene trees onto the coalescent species tree for all three pipelines to characterize the gene tree conflict against the phylogenetic signal in the nuclear phylogeny. Before mapping gene trees to species trees, all the trees were re-rooted using the outgroup. The PhyPartsPiecharts scripts developed by M. Johnson (https://github.com/mossmatters/phyloscripts/tree/master/phypartspiecharts) were used to summarize the results.

#### Network analysis

We inferred a phylogenetic network with SplitsTree v4.18.2 [45]. SplitsTree infers information from multiple loci and allows to represent evolutionary relationships with reticulation events, e.g., recombination, lateral gene transfer, and hybridization [46]. We used the alignment consisting of the concatenated nuclear loci for all three pipelines, excluding the outgroup species, with the settings: Jukes-Cantor for characters and NeighborNet methods for distances.

#### Morphological analysis

For the character state analyses, we extracted morphological data from the literature [48]. We coded 12 characters (leaf blade shape, leaf surface structure, stem shape, stem pubescence, petiole pubescence, calyx segment shape (male, female), calyx tube shape, corolla segment shape, anther shape, fruit shape, and ovary shape) as discrete characters. The analyses were performed using Mesquite v3.70 [47] with the parsimony model using the alignment consisting of the concatenated nuclear loci from the Captus pipeline.

### Electronic supplementary material

Below is the link to the electronic supplementary material.

Supplementary Material 1

Supplementary Material 2

Supplementary Material 3

Supplementary Material 4

Supplementary Material 5

Supplementary Material 6

Supplementary Material 7

Supplementary Material 8

Supplementary Material 9

Supplementary Material 10

Supplementary Material 11

Supplementary Material 12

Supplementary Material 13

Supplementary Material 14

Supplementary Material 15

Supplementary Material 16

Supplementary Material 17

Supplementary Material 18

Supplementary Material 19

Supplementary Material 20

Supplementary Material 21

Supplementary Material 22

Supplementary Material 23

Supplementary Material 24

## Data Availability

The raw Illumina data generated for this study are available through the Sequence Read Archive with BioProject PRJNA945336. The DNA alignments, configuration files, and major results are available via figshare https://figshare.com/. 10.6084/m9.figshare.22304314.v1. 10.6084/m9.figshare.22304539.v1. 10.6084/m9.figshare.22310149.v1. 10.6084/m9.figshare.22310170.v1. 10.6084/m9.figshare.22310179.v1. 10.6084/m9.figshare.22316767.v1. 10.6084/m9.figshare.22316833.v1. 10.6084/m9.figshare.22316911.v1. 10.6084/m9.figshare.22302910.v1. 10.6084/m9.figshare.22302937.v1. 10.6084/m9.figshare.22302952.v1. 10.6084/m9.figshare.22302994.v1. 10.6084/m9.figshare.22303087.v1. 10.6084/m9.figshare.22303327.v1. 10.6084/m9.figshare.22303708.v1. 10.6084/m9.figshare.22304107.v1. 10.6084/m9.figshare.22304197.v1. 10.6084/m9.figshare.22304281.v1. 10.6084/m9.figshare.22304296.v1.

## Abbreviations

SECAPR: Sequence Capture Processor
WGS: Whole genome sequencing
UFBS: Ultrafast bootstrap
ILS: Incomplete lineage sorting
ML: Maximum likelihood
AIC: Akaike information criterion
LPP: Local posterior probability
MSC: Multispecies coalescent
