# Supplementary figures and images for: Resolving the phylogeny of Thladiantha (Cucurbitaceae) with three different target capture pipelines
Source: BMC Ecol Evol. 2023 Dec 12;23:75. doi: 10.1186/s12862-023-02185-z (PMC10714463; doi:10.1186/s12862-023-02185-z)

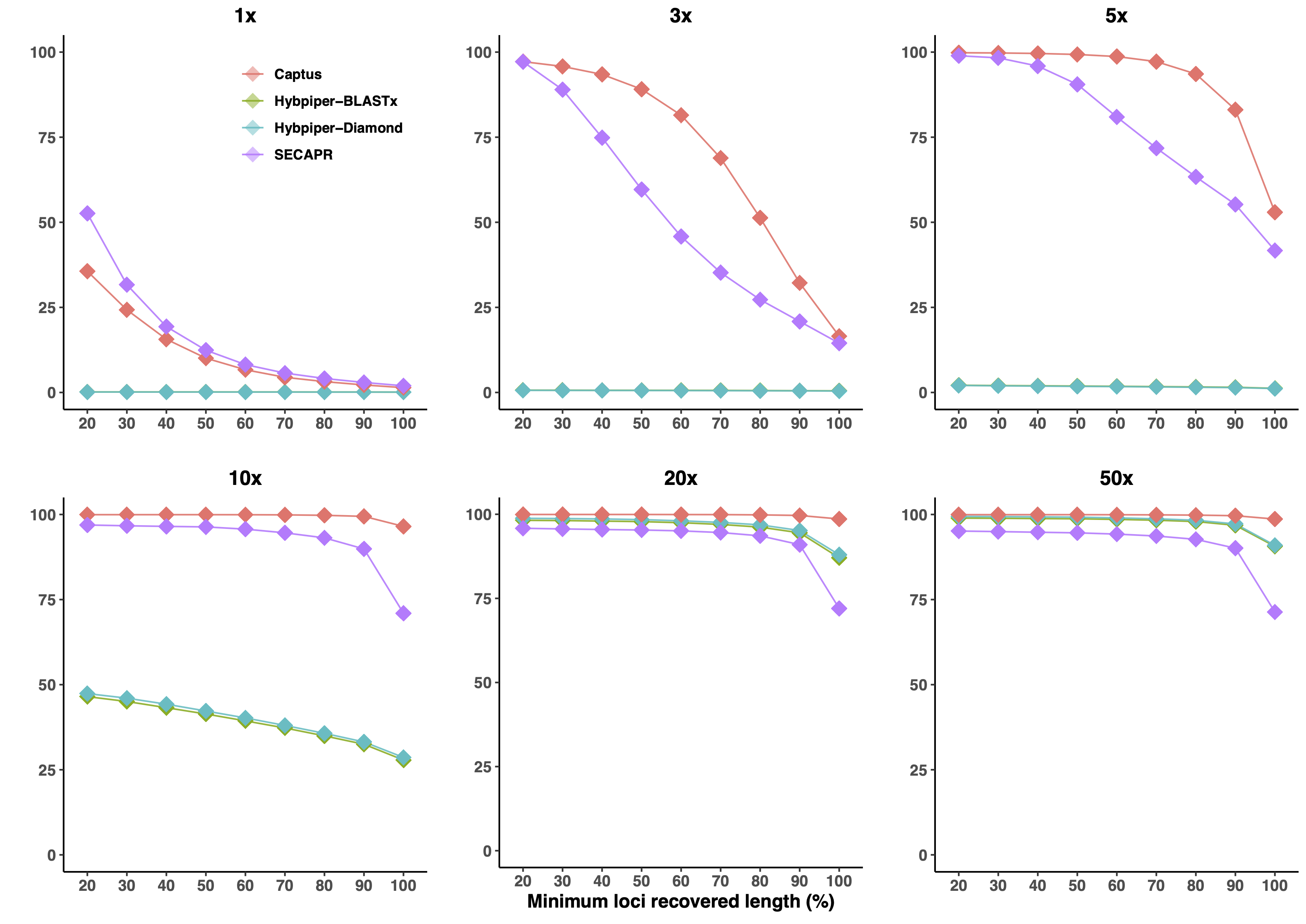

Supplement: Supplementary file 1 — Supplementary Material 1 [file 12862_2023_2185_MOESM1_ESM.png]

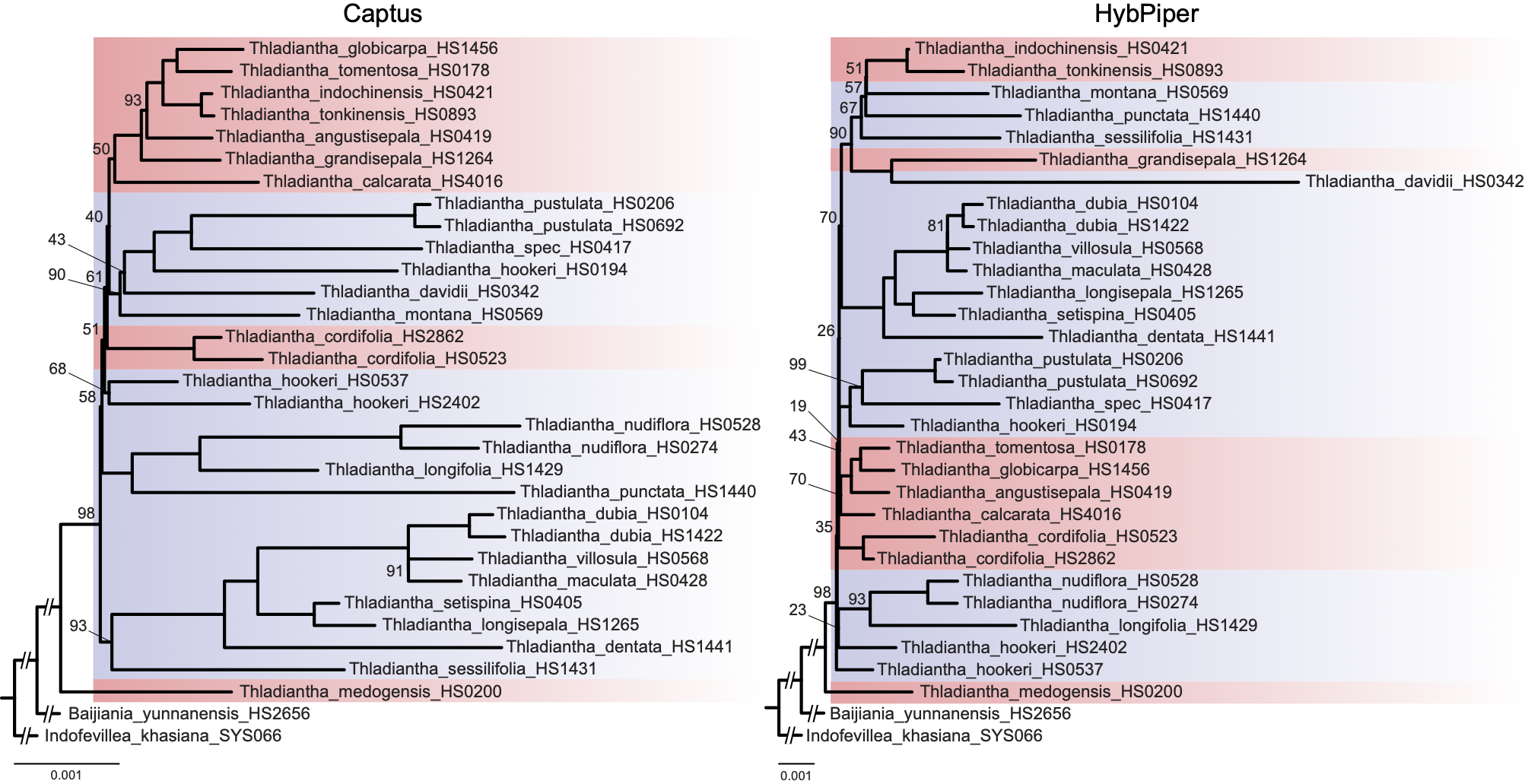

Supplement: Supplementary file 2 — Supplementary Material 2 [file 12862_2023_2185_MOESM2_ESM.png]

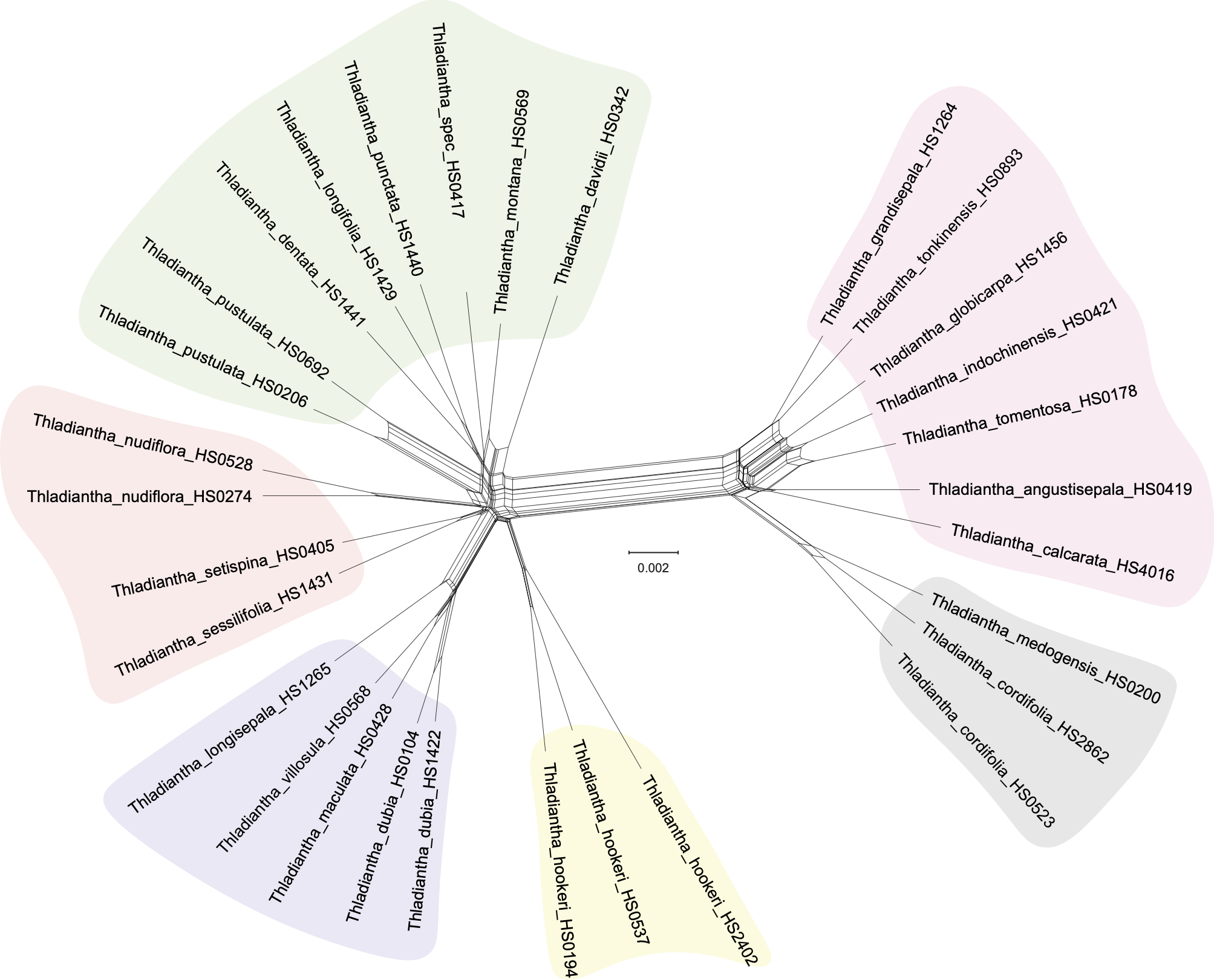

Supplement: Supplementary file 3 — Supplementary Material 3 [file 12862_2023_2185_MOESM3_ESM.png]

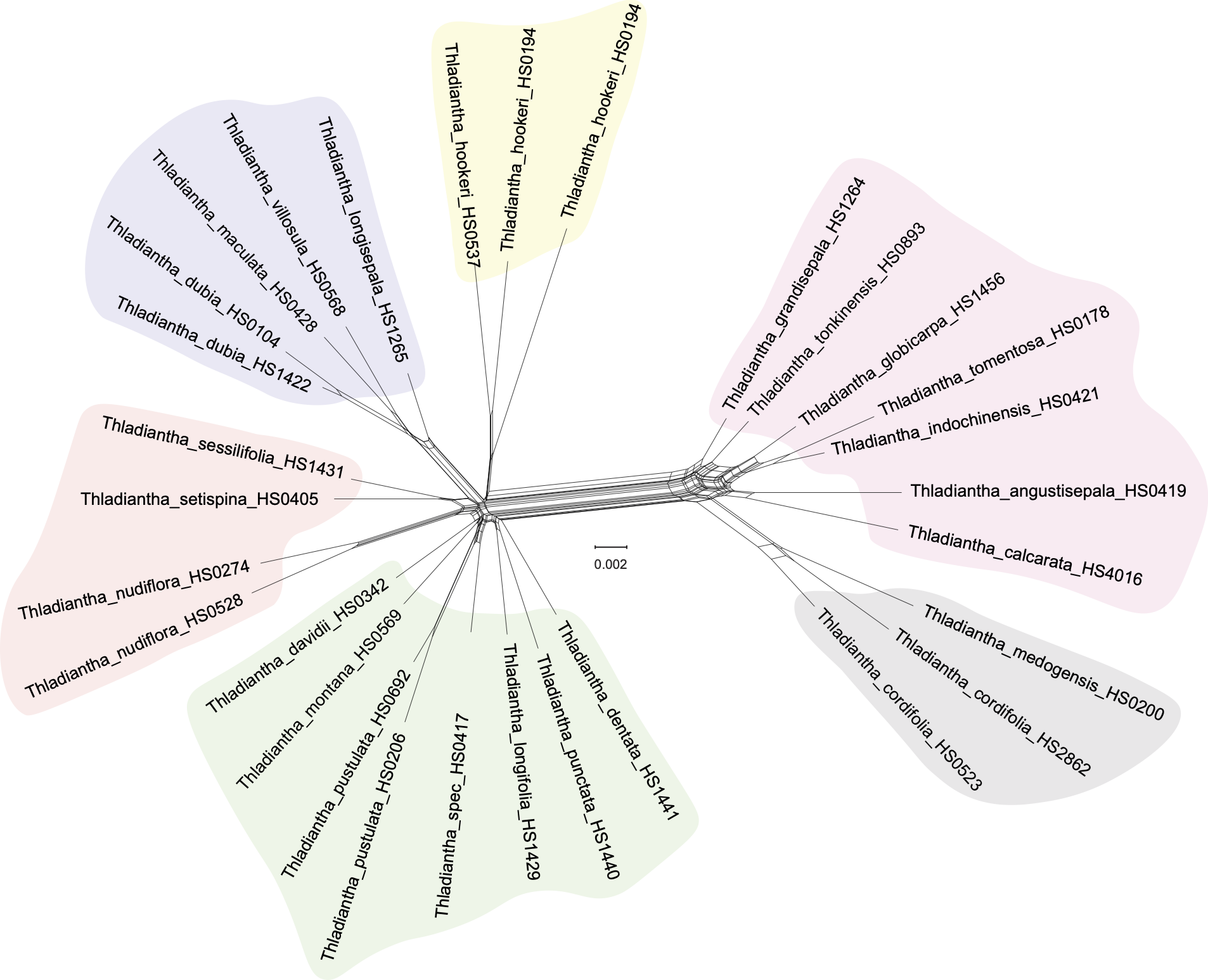

Supplement: Supplementary file 4 — Supplementary Material 4 [file 12862_2023_2185_MOESM4_ESM.png]

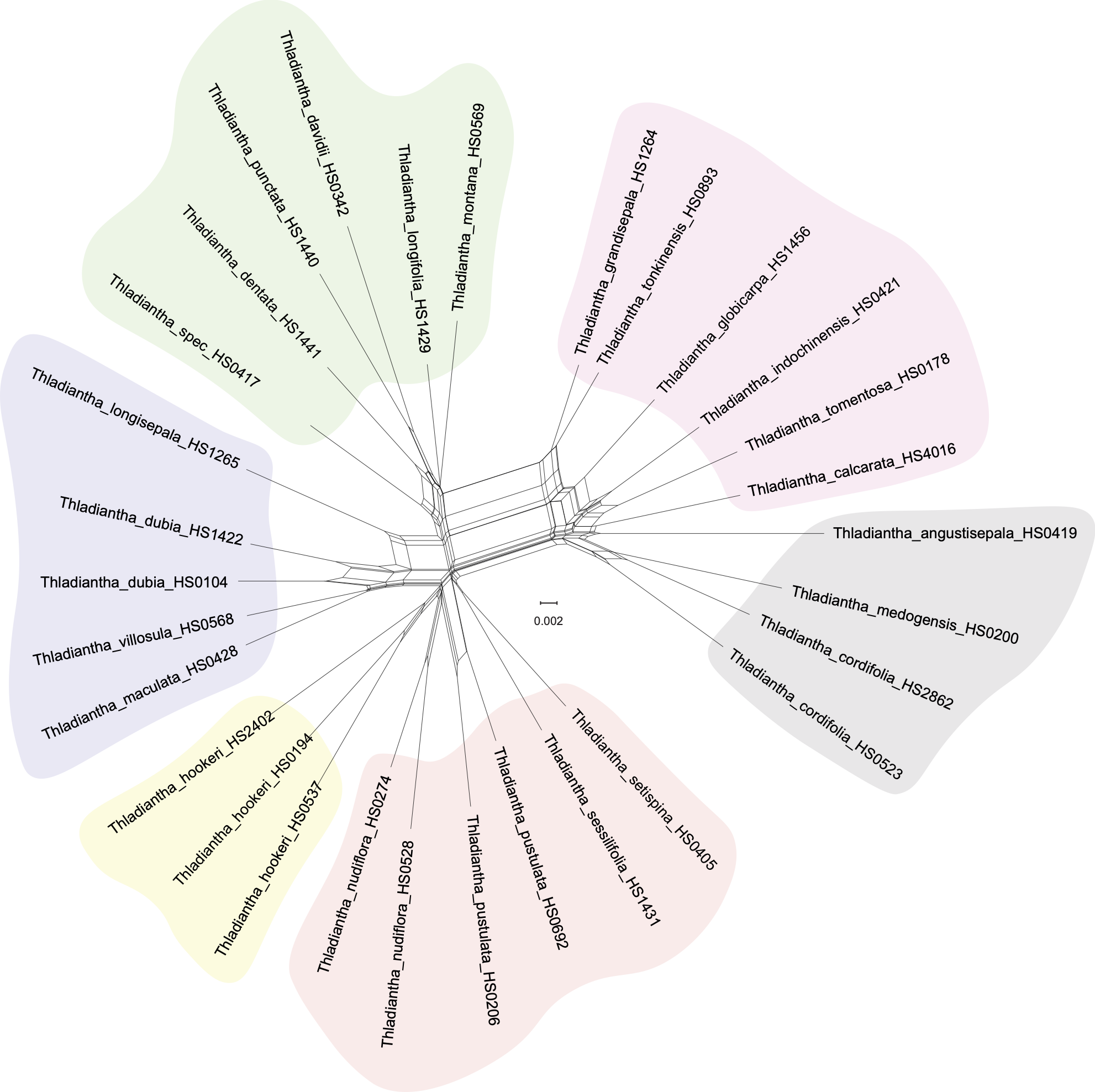

Supplement: Supplementary file 5 — Supplementary Material 5 [file 12862_2023_2185_MOESM5_ESM.png]

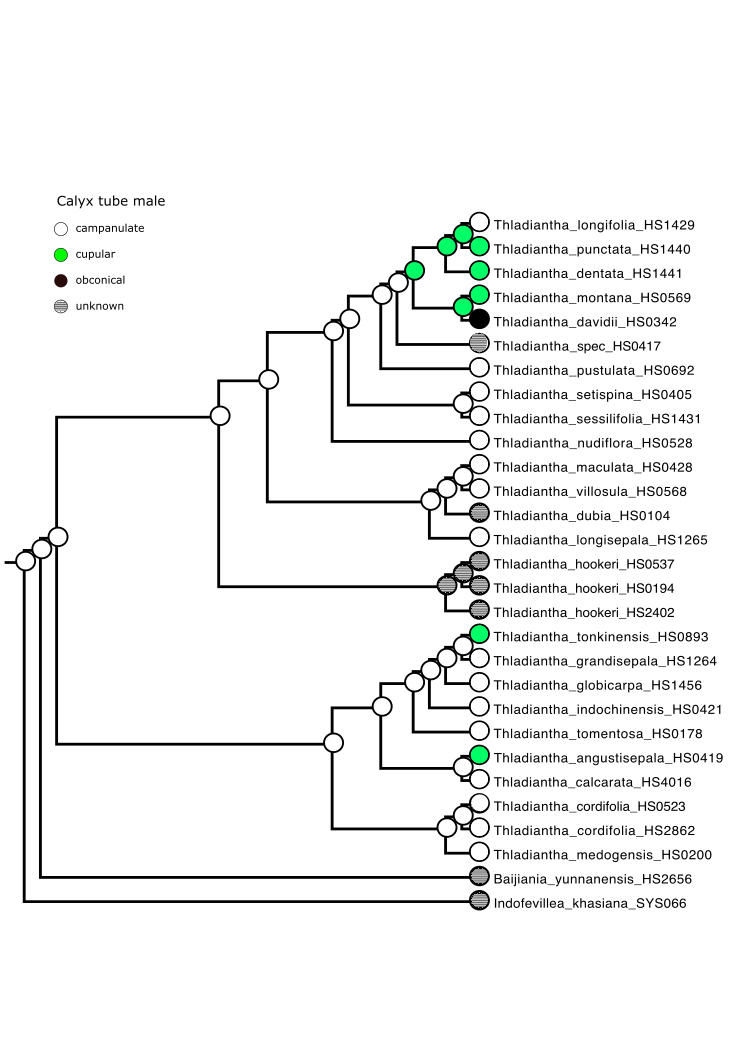

Supplement: Supplementary file 6 — Supplementary Material 6 [file 12862_2023_2185_MOESM6_ESM.png]

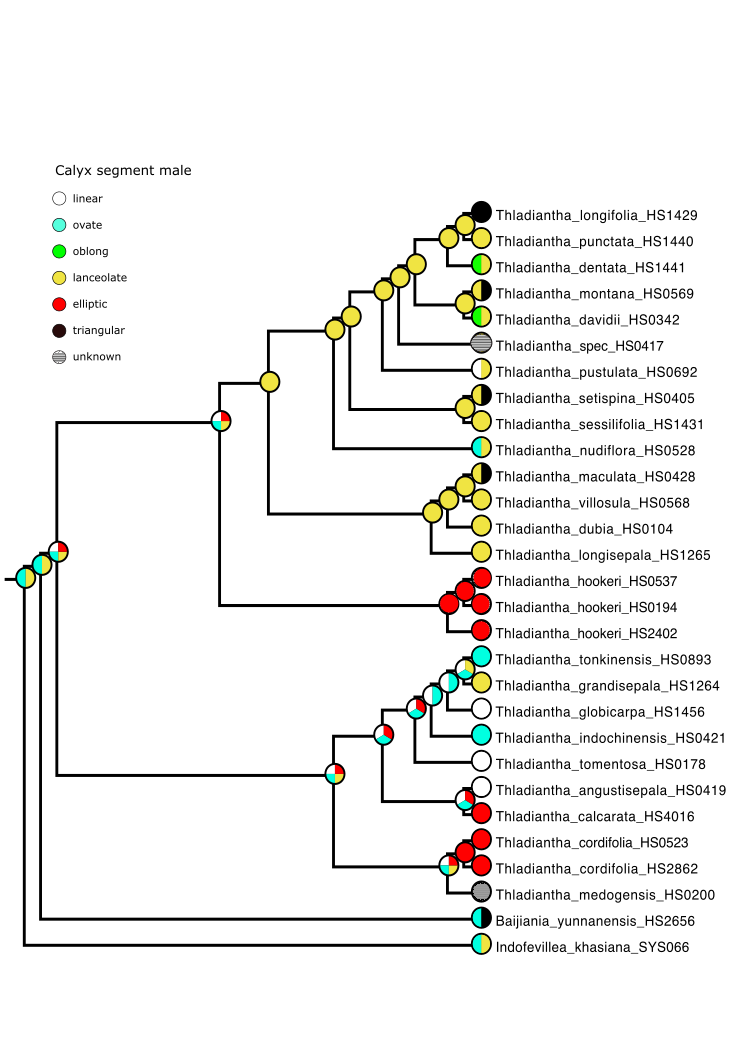

Supplement: Supplementary file 7 — Supplementary Material 7 [file 12862_2023_2185_MOESM7_ESM.png]

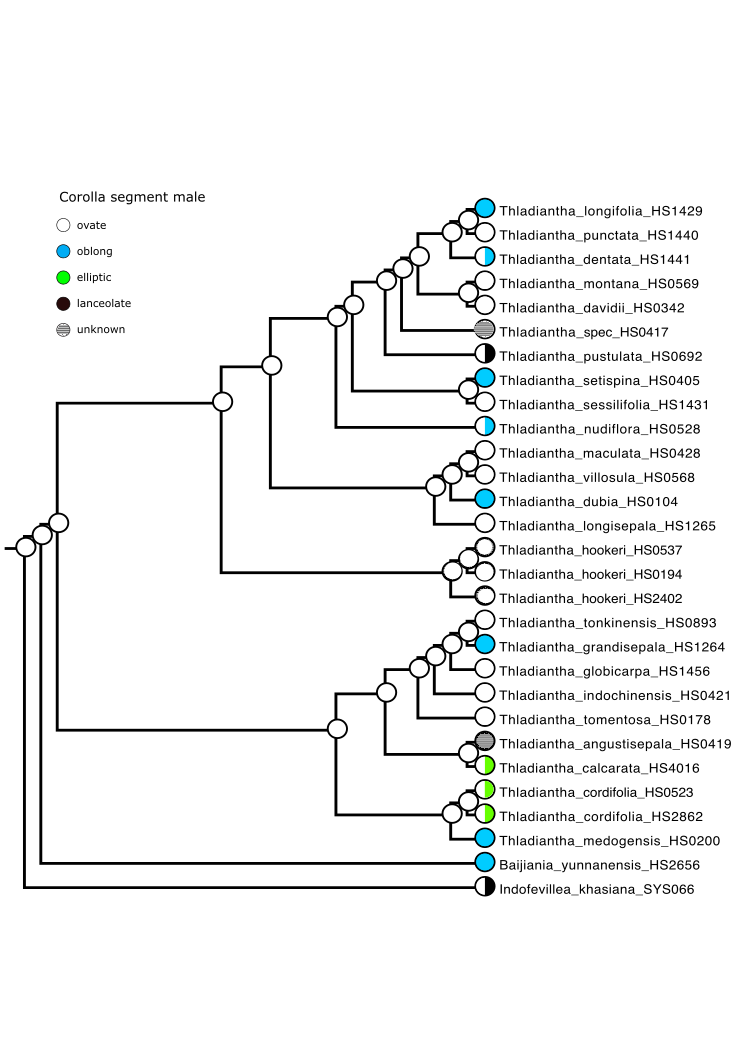

Supplement: Supplementary file 8 — Supplementary Material 8 [file 12862_2023_2185_MOESM8_ESM.png]

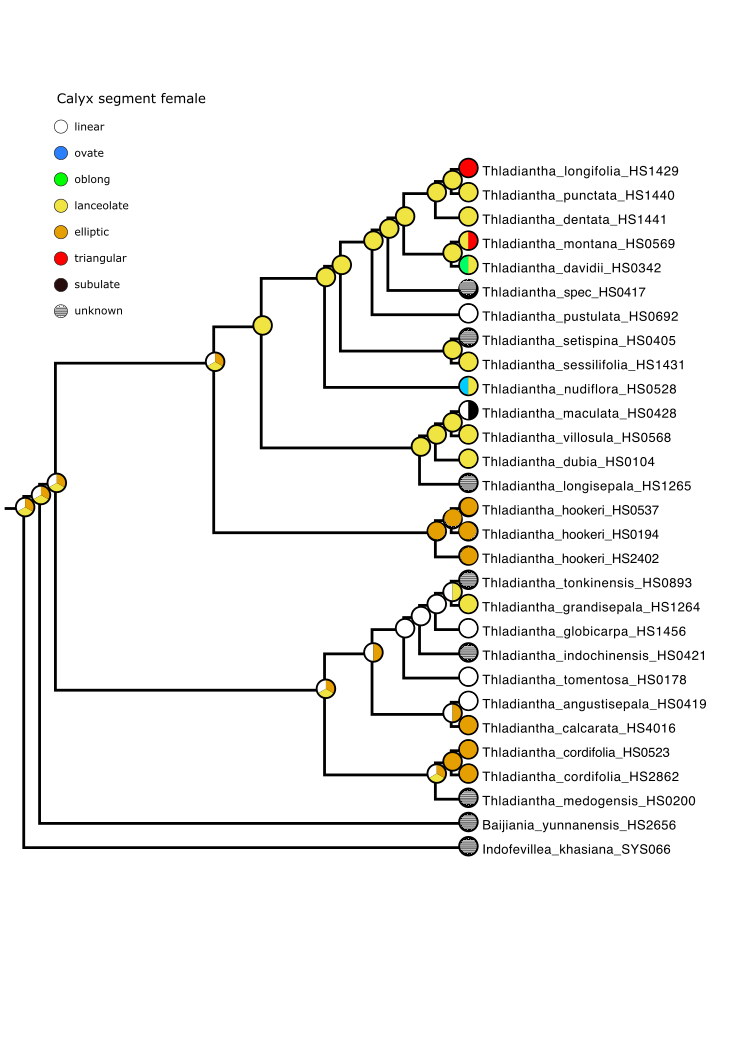

Supplement: Supplementary file 9 — Supplementary Material 9 [file 12862_2023_2185_MOESM9_ESM.png]

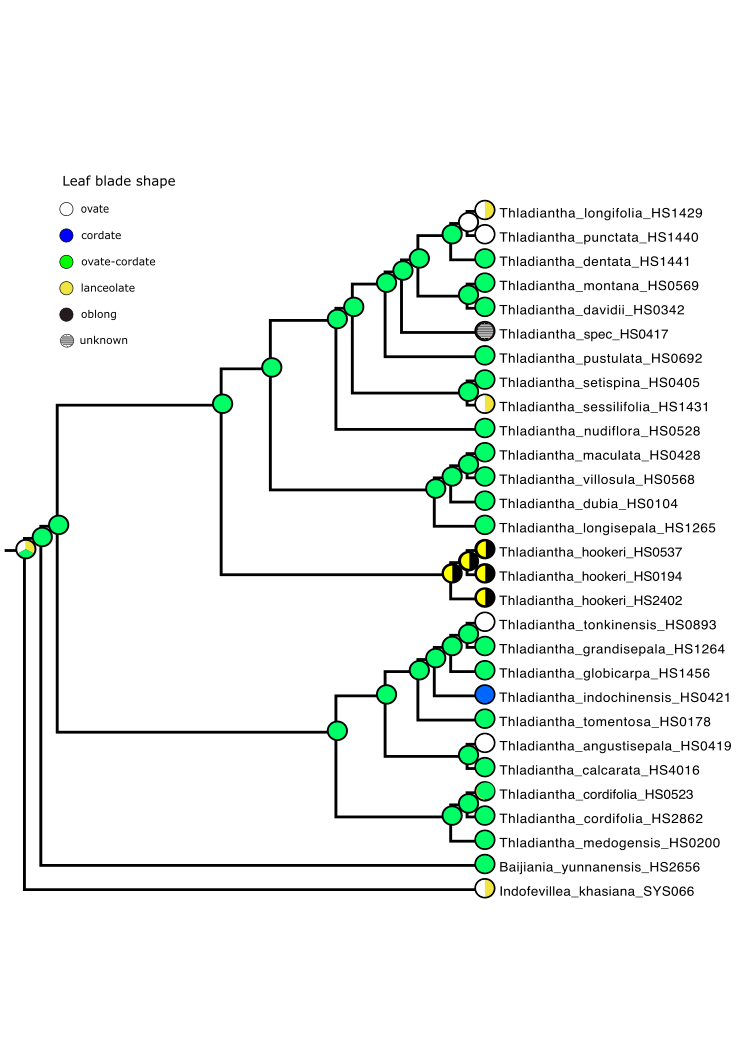

Supplement: Supplementary file 10 — Supplementary Material 10 [file 12862_2023_2185_MOESM10_ESM.png]

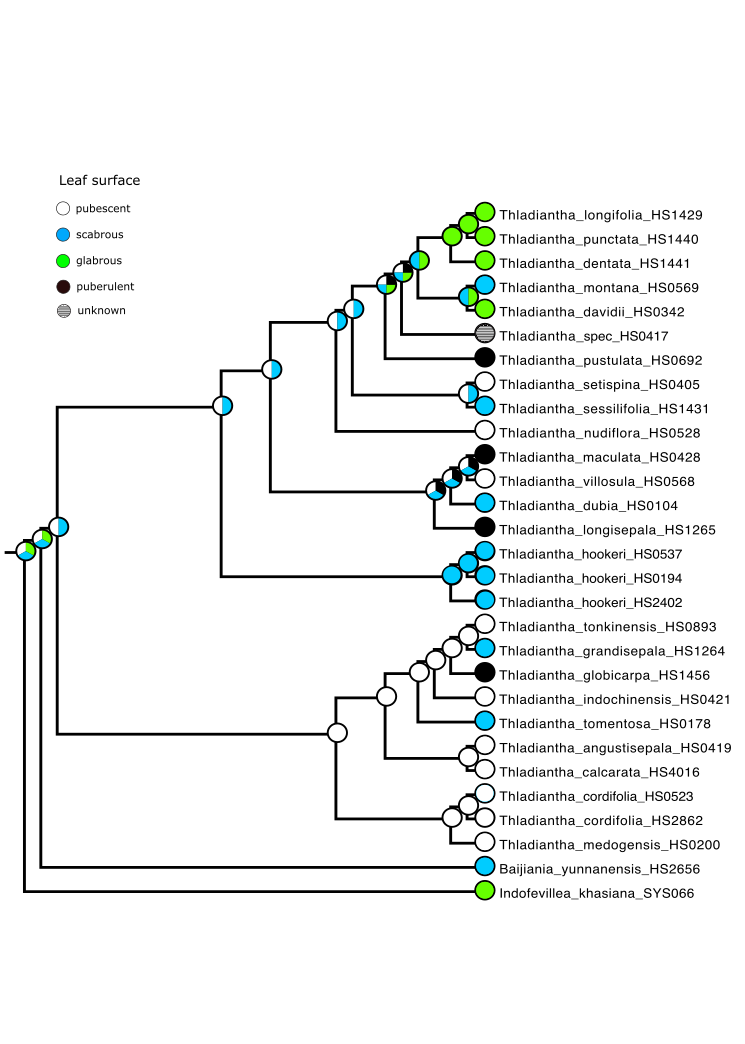

Supplement: Supplementary file 11 — Supplementary Material 11 [file 12862_2023_2185_MOESM11_ESM.png]

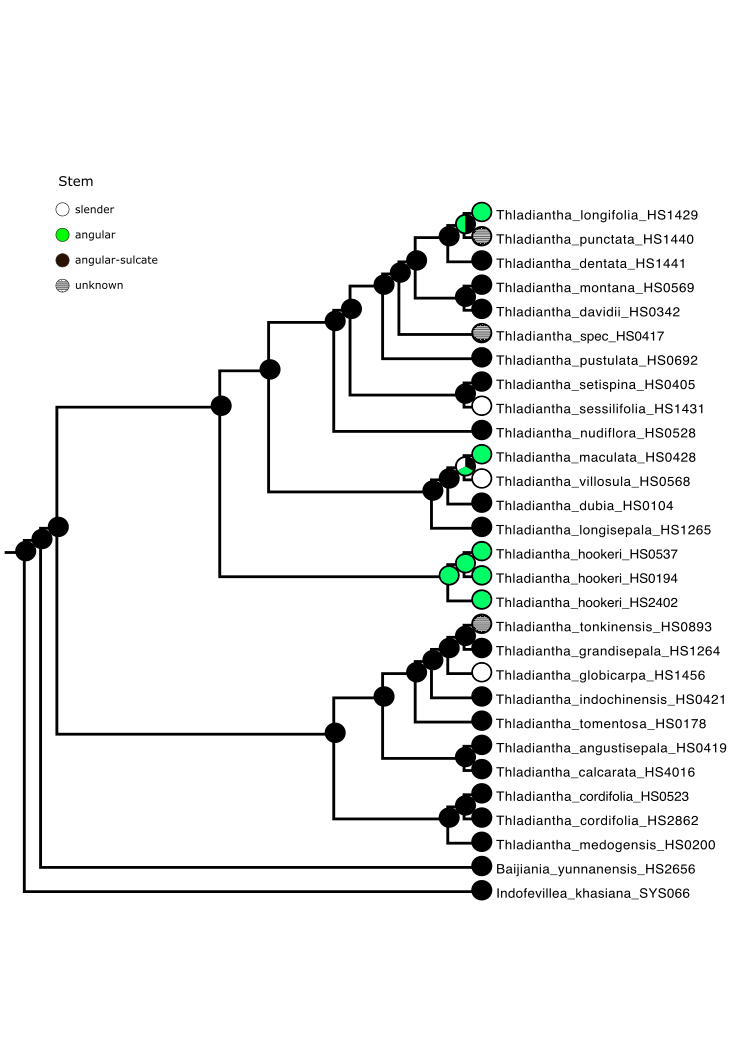

Supplement: Supplementary file 12 — Supplementary Material 12 [file 12862_2023_2185_MOESM12_ESM.png]

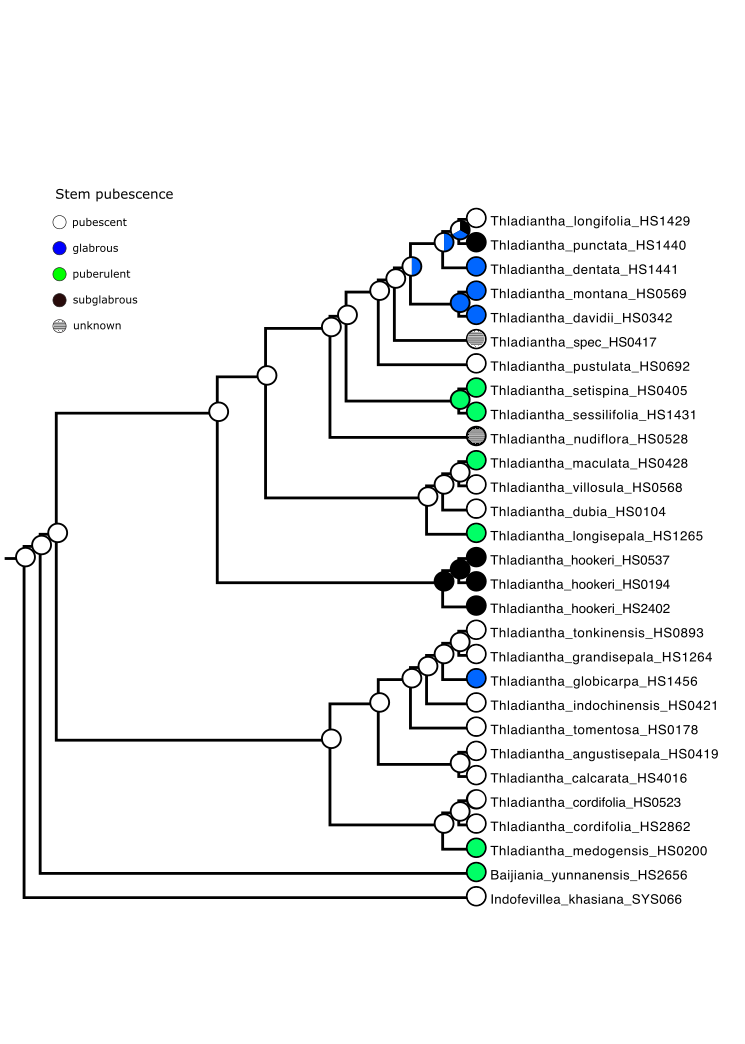

Supplement: Supplementary file 13 — Supplementary Material 13 [file 12862_2023_2185_MOESM13_ESM.png]

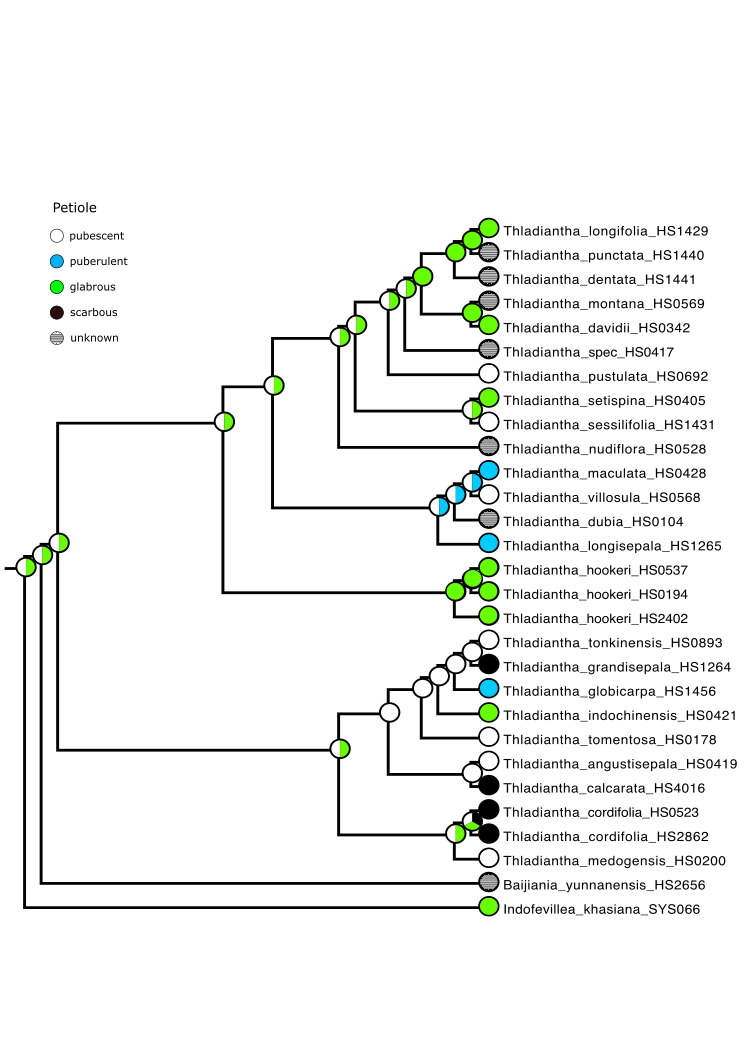

Supplement: Supplementary file 14 — Supplementary Material 14 [file 12862_2023_2185_MOESM14_ESM.png]

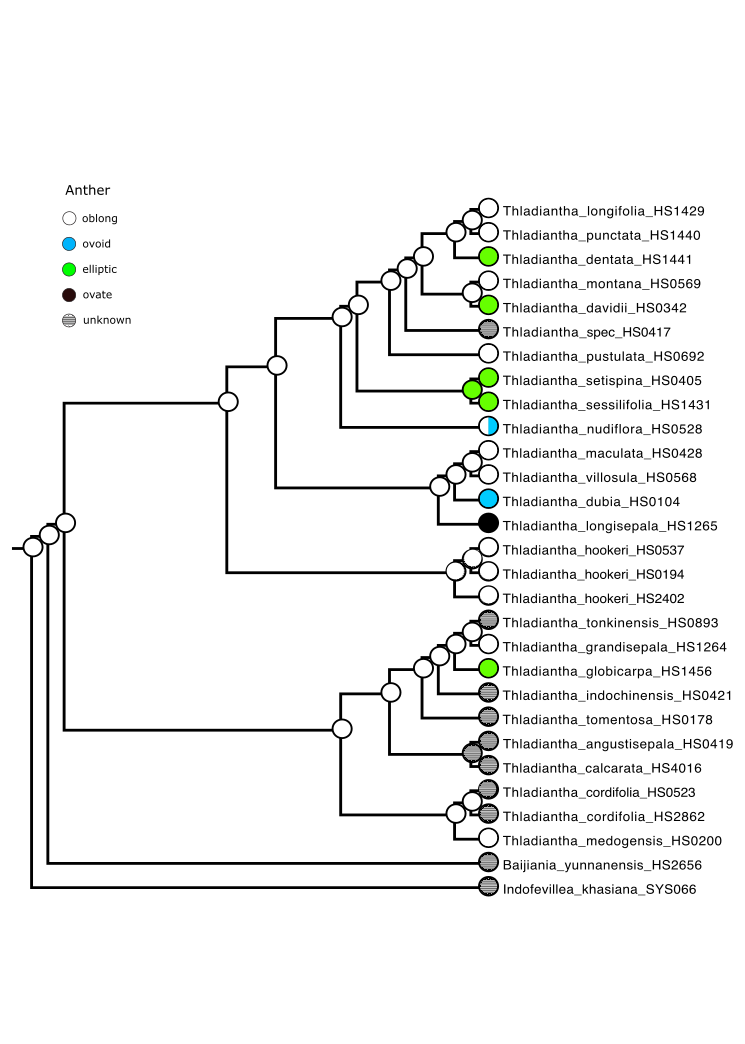

Supplement: Supplementary file 15 — Supplementary Material 15 [file 12862_2023_2185_MOESM15_ESM.png]

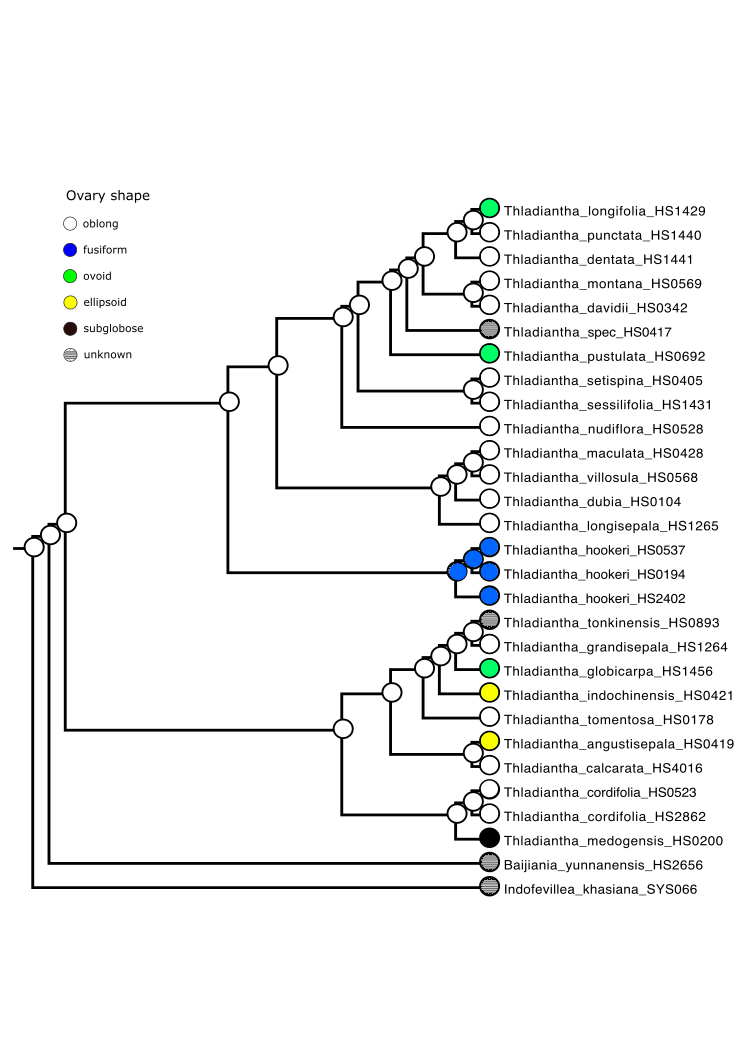

Supplement: Supplementary file 16 — Supplementary Material 16 [file 12862_2023_2185_MOESM16_ESM.png]

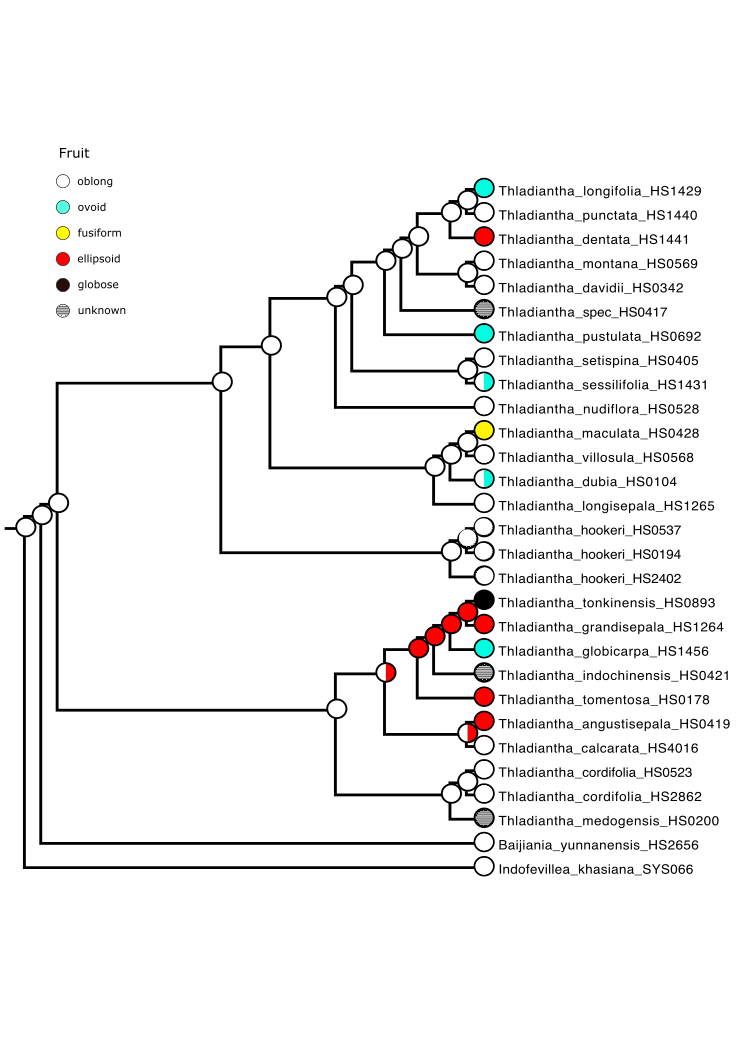

Supplement: Supplementary file 17 — Supplementary Material 17 [file 12862_2023_2185_MOESM17_ESM.png]

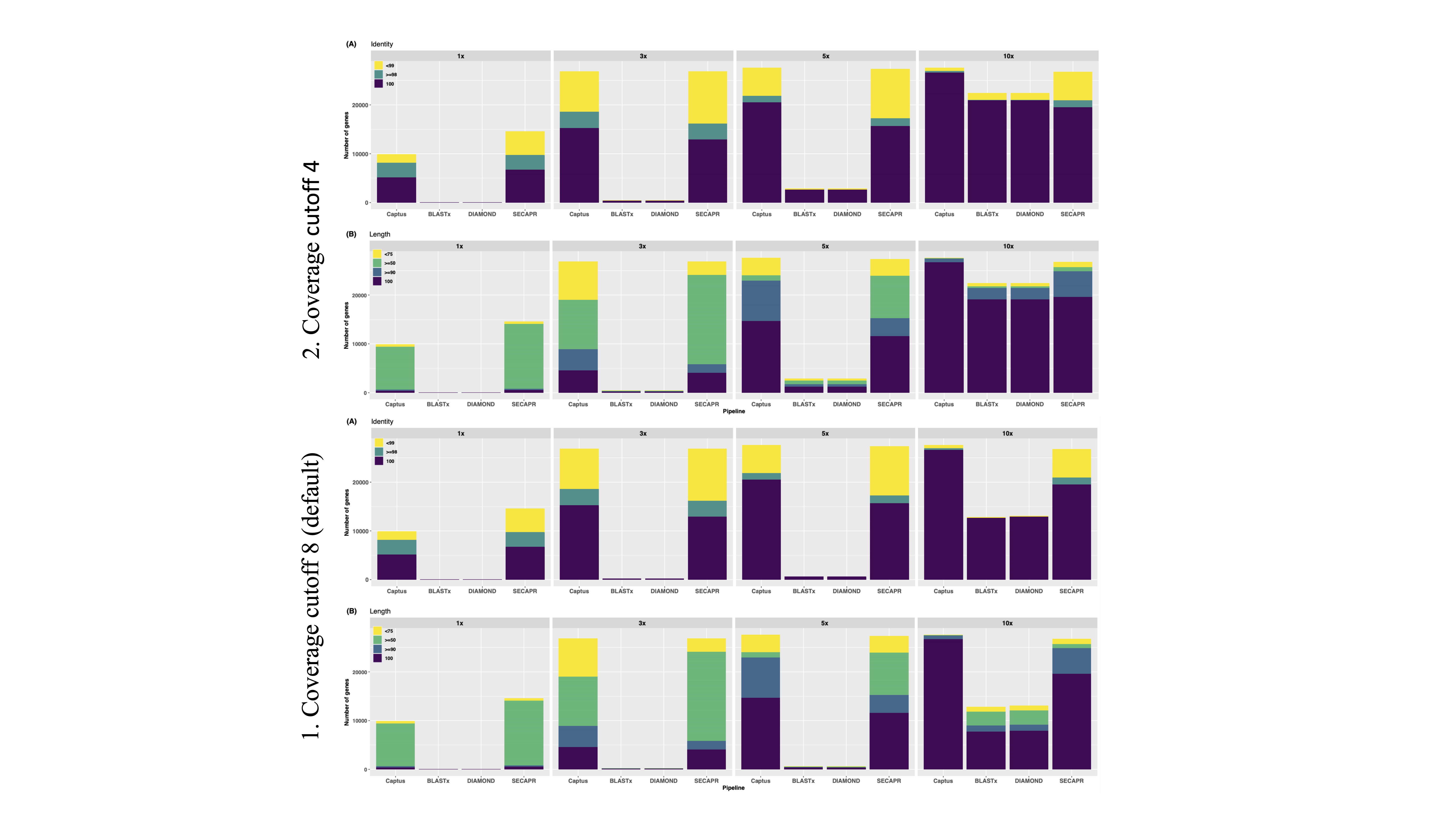

Supplement: Supplementary file 18 — Supplementary Material 18 [file 12862_2023_2185_MOESM18_ESM.png]
