# Supplementary material for: Resolving the phylogeny of Thladiantha (Cucurbitaceae) with three different target capture pipelines
Source: BMC Ecol Evol. 2023 Dec 12;23:75. doi: 10.1186/s12862-023-02185-z (PMC10714463; doi:10.1186/s12862-023-02185-z)
Supplement: Supplementary file 19 — Supplementary Material 19 [file 12862_2023_2185_MOESM19_ESM.docx]

**Table S1: Table of material with voucher information for all specimens**

| DNA No. | Species | Origin | Voucher |
| --- | --- | --- | --- |
| HS0104 | *Thladiantha dubia* | cult. BG Munich | H. Schaefer s.n. |
| HS1422 | *Thladiantha dubia* | Russia | S. Kharkevich & T. Buch 272 (NY) |
| HS0178 | *Thladiantha tomentosa* | Yunnan, China | H. Schaefer 2005/127 (M) |
| HS0194 | *Thladiantha hookeri* | Yunnan, China | H. Schaefer 2005/120 (M) |
| HS0200 | *Thladiantha medogensis* | Medog, Tibet | (KUN 0370549) |
| HS0206 | *Thladiantha pustulata* | Yunnan, China | H. Schaefer 2005/121 (M) |
| HS0692 | *Thladiantha pustulata* | Guangdong, China | H. Schaefer  05/635 (M) |
| HS0274 | *Thladiantha nudiflora* | Mindanao, Philippines | W. Schwabe s.n. (B) |
| HS0528 | *Thladiantha nudiflora* | Anhui,  China | S. Fan & Y. Li 77 (CAS) |
| HS0342 | *Thladiantha davidii* | China | (IBSC 79) |
| HS0417 | *Thladiantha spec.* | China | (WU) |
| HS0419 | *Thladiantha angustisepala* | Thailand | W. J.J. De Wilde & B. Duyfjes 5204 (L) |
| HS0421 | *Thladiantha indochinensis* | Vietnam | Petelot 2197 (A) |
| HS0428 | *Thladiantha maculata* | Hubei China | B. Bartholomew et al. 1323 (A) |
| HS0523 | *Thladiantha cordifolia* | Thailand | J. Maxwell 93-890 (A) |
| HS0537 | *Thladiantha* *hookeri* | China | C. W. Wang 68162 (A) |
| HS0568 | *Thladiantha villosula* | China | (BR) |
| HS0569 | *Thladiantha montana* | China | (BR) |
| HS0405 | *Thladiantha setispina* | China | Cunningham 219 (E) |
| HS0893 | *Thladiantha tonkinensis* | Vietnam | B. Balansa 4549 (US) |
| HS1264 | *Thladiantha grandisepala* | China | C. W. Wang 77327 (GH) |
| HS1265 | *Thladiantha longisepala* |  | C. W. Wang 63877 (GH) |
| HS1429 | *Thladiantha longifolia* | W Hubei,  China | E. H. Wilson 2271 (NY) |
| HS1431 | *Thladiantha sessilifolia* | Tibet | R. R. Soulie (NY)  det. *T. nudiflora*, rev. *T. setispina* |
| HS1440 | *Thladiantha punctata* | Guangdong,  China | C. L. Tso 20513 (NY) |
| HS1441 | *Thladiantha dentata* | Hubei, China | Ho Ch'ang Chow 305 (NY) |
| HS1456 | *Thladiantha globicarpa* | Hunan,  China | Luo Liu-bo 0131 (NY) |
| HS2402 | *Thladiantha hookeri* | Kachin,  Myanmar | H. Schaefer, D. A. Santamaria & D. E. Boufford 121 (MAND) |
| HS2862 | *Thladiantha cordifolia* | Chin State,  Myanmar | Ling Shein Man et al. 054002 (TUM) |
| HS4016 | *Thladiantha calcarata* | Kachin,  Myanmar | cultivated at Freising  HSC64 |
| HS2656 | *Baijiania yunnanensis* | unknown | cultivated at Freising HSC73 |
| SYS066 | *Indofevillea khasiana* | Kachin, Myanmar | H. Schaefer, D.A. Santamaria & D.E. Boufford 154 (MAND) |
