# Supplementary material for: Resolving the phylogeny of Thladiantha (Cucurbitaceae) with three different target capture pipelines
Source: BMC Ecol Evol. 2023 Dec 12;23:75. doi: 10.1186/s12862-023-02185-z (PMC10714463; doi:10.1186/s12862-023-02185-z)
Supplement: Supplementary file 20 — Supplementary Material 20 [file 12862_2023_2185_MOESM20_ESM.docx]

**Table S2: Computation time (cumulative) comparison of three different pipelines**

| Pipeline | Assembly time | Extraction time |
| --- | --- | --- |
| HybPiper-BLASTx | 19 hr 55 min 30 sec | 19 hr 2 min 15 sec |
| HybPiper-DIAMOND | 24 hr 3 min 29 sec | 8 hr 59 min 28 sec |
| SECAPR | 8 hr 6 min 58 sec | 6 min 23 sec |
| Captus | 1 hr 23 min 21 sec | 34 min 15 sec |
