# Supplementary material for: Resolving the phylogeny of Thladiantha (Cucurbitaceae) with three different target capture pipelines
Source: BMC Ecol Evol. 2023 Dec 12;23:75. doi: 10.1186/s12862-023-02185-z (PMC10714463; doi:10.1186/s12862-023-02185-z)
Supplement: Supplementary file 21 — Supplementary Material 21 [file 12862_2023_2185_MOESM21_ESM.docx]

**Table S3: Computation time (cumulative) comparison of three different pipelines in *Arabidopsis thaliana.***

| Pipeline | Assembly time | Extraction time |
| --- | --- | --- |
| HybPiper-BLASTx | 5 hr 8 min 46 sec | 10 hr 44 min 48 sec |
| HybPiper-DIAMOND | 4 hr 20 min 7 sec | 3 hr 26 min 38 sec |
| SECAPR | 2 hr 54 min 22 sec | 5 hr 3 min 5 sec |
| Captus | 58 min 55 sec | 60 min 40 sec |
