# Supplementary material for: Resolving the phylogeny of Thladiantha (Cucurbitaceae) with three different target capture pipelines
Source: BMC Ecol Evol. 2023 Dec 12;23:75. doi: 10.1186/s12862-023-02185-z (PMC10714463; doi:10.1186/s12862-023-02185-z)
Supplement: Supplementary file 22 — Supplementary Material 22 [file 12862_2023_2185_MOESM22_ESM.docx]

**Table S4. Sample-wise comparison of three pipelines including clean reads, no. of recovered genes, mean recovery of the genes, and sd.**

|  | **Clean Reads** | **HybPiper-BLASTx** | | | **HybPiper-DIAMOND** | | | **SECAPR** | | | **Captus** | | |
| --- | --- | --- | --- | --- | --- | --- | --- | --- | --- | --- | --- | --- | --- |
|  |  | **Recovered genes** | **Mean recovery** | **SD** | **Recovered genes** | **Mean recovery** | **SD** | **Recovered genes** | **Mean recovery** | **SD** | **Recovered genes** | **Mean recovery** | **SD** |
| *Thladiantha angustisepala HS0419* | 9725698 | 1141 | 94.02 | 12.62 | 1138 | 93.73 | 13.02 | 1164 | 86.07 | 18.63 | 1176 | 94.47 | 11.92 |
| *Thladiantha* calcarata HS4016 | 4145486 | 1110 | 91.91 | 15.33 | 1110 | 91.81 | 15.66 | 1165 | 83.72 | 20.43 | 1172 | 92.72 | 13.72 |
| *Thladiantha* tomentosa HS0178 | 4645078 | 1128 | 94.44 | 13.49 | 1125 | 94.63 | 13.36 | 1164 | 89.01 | 17.47 | 1176 | 95.63 | 10.94 |
| *Thladiantha* cordifolia HS2862 | 6476292 | 1119 | 93.55 | 14.20 | 1123 | 93.62 | 13.91 | 1166 | 87.00 | 18.11 | 1170 | 95.35 | 10.47 |
| *Thladiantha* davidii HS0342 | 105478 | 469 | 46.42 | 20.59 | 468 | 45.85 | 20.51 | 520 | 38.59 | 15.77 | 597 | 48.24 | 19.31 |
| *Thladiantha* dentata HS1441 | 2708472 | 867 | 70.95 | 24.34 | 868 | 69.63 | 24.34 | 866 | 53.98 | 24.83 | 994 | 70.12 | 23.95 |
| *Thladiantha* spec. HS0417 | 357030 | 923 | 78.69 | 23.76 | 917 | 78.29 | 23.71 | 1038 | 64.13 | 26.74 | 1026 | 79.22 | 22.44 |
| *Thladiantha* dubia HS0104 | 2335796 | 1086 | 93.88 | 13.62 | 1086 | 93.82 | 13.64 | 1163 | 87.65 | 18.21 | 1163 | 94.27 | 12.44 |
| *Thladiantha* dubia HS1422 | 2508378 | 1076 | 87.82 | 19.46 | 1078 | 87.70 | 19.64 | 1145 | 78.34 | 23.36 | 1151 | 88.44 | 18.13 |
| *Thladiantha* globicarpa HS1456 | 2494010 | 1038 | 85.72 | 21.19 | 1037 | 85.59 | 21.36 | 1131 | 73.81 | 25.24 | 1129 | 86.30 | 19.44 |
| *Thladiantha* grandisepala HS1264 | 3540708 | 934 | 72.40 | 24.80 | 936 | 71.57 | 24.62 | 914 | 54.90 | 25.41 | 1005 | 73.11 | 23.21 |
| *Thladiantha* hookeri HS0194 | 1791650 | 1069 | 91.69 | 16.15 | 1068 | 91.57 | 16.32 | 1162 | 85.71 | 19.44 | 1164 | 93.01 | 14.13 |
| *Thladiantha* cordifolia HS0523 | 1520582 | 1098 | 86.38 | 20.16 | 1095 | 86.29 | 20.19 | 1149 | 73.34 | 25.08 | 1153 | 87.04 | 18.89 |
| *Thladiantha* indochinensis HS0421 | 2061678 | 1100 | 88.54 | 18.68 | 1107 | 88.50 | 18.66 | 1159 | 77.90 | 23.27 | 1162 | 88.91 | 17.54 |
| *Thladiantha* longifolia HS1429 | 5109440 | 1011 | 75.03 | 24.06 | 1015 | 73.96 | 24.34 | 958 | 56.62 | 25.84 | 1074 | 76.03 | 22.85 |
| *Thladiantha* longisepala HS1265 | 1543550 | 950 | 82.75 | 22.42 | 956 | 82.12 | 22.69 | 1099 | 68.66 | 26.34 | 1102 | 83.11 | 21.58 |
| *Thladiantha* maculata HS0428 | 7050292 | 1144 | 94.90 | 11.86 | 1144 | 94.83 | 11.90 | 1166 | 87.39 | 17.89 | 1175 | 96.12 | 09.76 |
| *Thladiantha* medogensis HS0200 | 6080422 | 1126 | 95.00 | 12.54 | 1130 | 94.98 | 12.55 | 1163 | 89.84 | 16.51 | 1168 | 96.04 | 10.22 |
| *Thladiantha* montana HS0569 | 1452534 | 1055 | 77.90 | 23.74 | 1056 | 77.34 | 23.59 | 1013 | 60.07 | 26.32 | 1069 | 77.50 | 23.21 |
| *Thladiantha* nudiflora HS0274 | 3222464 | 1127 | 91.51 | 15.30 | 1124 | 91.68 | 15.21 | 1164 | 82.65 | 20.96 | 1164 | 92.45 | 13.91 |
| *Thladiantha* nudiflora HS0528 | 6696398 | 1134 | 93.35 | 13.15 | 1135 | 93.39 | 13.09 | 1165 | 85.13 | 19.93 | 1174 | 93.84 | 12.01 |
| *Thladiantha* punctata HS1440 | 1642230 | 751 | 67.26 | 24.72 | 756 | 66.12 | 24.41 | 808 | 51.60 | 23.89 | 897 | 66.96 | 24.03 |
| *Thladiantha* pustulata HS0206 | 7702348 | 1137 | 94.90 | 11.96 | 1137 | 95.05 | 11.76 | 1166 | 88.00 | 17.79 | 1174 | 95.33 | 10.87 |
| *Thladiantha* pustulata HS0692 | 2180424 | 978 | 86.32 | 20.88 | 979 | 86.02 | 21.10 | 1126 | 75.76 | 24.74 | 1126 | 86.73 | 19.56 |
| *Thladiantha* sessilifolia HS1431 | 6987084 | 1128 | 86.10 | 19.60 | 1125 | 86.11 | 19.37 | 1151 | 72.74 | 25.29 | 1159 | 86.78 | 18.14 |
| *Thladiantha* setispina HS0405 | 2508782 | 1134 | 88.85 | 18.33 | 1133 | 89.07 | 17.92 | 1162 | 78.52 | 23.20 | 1162 | 90.14 | 16.17 |
| *Thladiantha* hookeri HS0537 | 7604168 | 1143 | 93.04 | 14.24 | 1143 | 93.18 | 14.14 | 1167 | 85.70 | 19.36 | 1172 | 94.46 | 11.77 |
| *Thladiantha* hookeri HS2402 | 3865514 | 1098 | 91.53 | 15.99 | 1095 | 91.35 | 16.35 | 1165 | 83.50 | 20.75 | 1167 | 92.22 | 14.69 |
| *Thladiantha* tonkinensis HS0893 | 4129798 | 956 | 73.83 | 24.43 | 960 | 73.39 | 24.21 | 930 | 56.60 | 25.76 | 1055 | 73.94 | 23.61 |
| *Thladiantha* villosula HS0568 | 5206034 | 1150 | 92.15 | 15.10 | 1149 | 92.07 | 15.37 | 1168 | 84.09 | 20.35 | 1168 | 92.18 | 13.13 |
| Baijiania yunnanensis HS2656 | 9896730 | 48 | 67.61 | 27.74 | 187 | 98.29 | 5.35 | 1139 | 64.46 | 22.94 | 1180 | 90.65 | 10.83 |
| Indofevillea khasiana SYS066 | 9136606 | 13 | 96.81 | 9.7 | 114 | 98.23 | 5.80 | 982 | 55.80 | 22.99 | 1164 | 87.10 | 13.11 |
