# Supplementary material for: Resolving the phylogeny of Thladiantha (Cucurbitaceae) with three different target capture pipelines
Source: BMC Ecol Evol. 2023 Dec 12;23:75. doi: 10.1186/s12862-023-02185-z (PMC10714463; doi:10.1186/s12862-023-02185-z)
Supplement: Supplementary file 24 — Supplementary Material 24 [file 12862_2023_2185_MOESM24_ESM.docx]

**Supplementary Information**

Additional File 1: Figure S1.png

Exon recovery length at different sequencing depths for simulated *Arabidopsis thaliana* reads with three pipelines. Recovered loci are sorted in bins ranging from 20% to 100% recovered length, as the minimum coverage threshold was 20.

Additional File 1: Figure S2.png

Plastome phylogeny of *Thladianta* using Captus and HybPiper. According to nuclear phylogeny, blue represents section 1, and red represents section 2.

Additional File 1: Figure S3.png

Phylogenetic network of *Thladiantha* inferred with SplitsTree using the concatenated alignment of Captus using Neighbor Net.

Additional File 1: Figure S4.png

Phylogenetic network of *Thladiantha* inferred with SplitsTree using the concatenated alignment of HybPiper using Neighbor Net.

Additional File 1: Figure S5.png

Phylogenetic network of *Thladiantha* inferred with SplitsTree using the concatenated alignment of SECAPR using Neighbor Net.

Additional File 1: Figure S6.png

Ancestral character state of calyx tube male.

Additional File 1: Figure S7.png

Ancestral character state of calyx segment male.

Additional File 1: Figure S8.png

Ancestral character state of corolla segment male.

Additional File 1: Figure S9.png

Ancestral character state of calyx segment female.

Additional File 1: Figure S10.png

Ancestral character state of leaf blade shape.

Additional File 1: Figure S11.png

Ancestral character state of leaf surface.

Additional File 1: Figure S12.png

Ancestral character state of stem.

Additional File 1: Figure S13.png

Ancestral character state of stem pubescence.

Additional File 1: Figure S14.png

Ancestral character state of petiole.

Additional File 1: Figure S15.png

Ancestral character state of anther.

Additional File 1: Figure S16.png

Ancestral character state of ovary shape.

Additional File 1: Figure S17.png

Ancestral character state of fruit shape.

Additional File 1: Figure S18.png

Identity and length recovery at different sequencing depths for simulated *Arabidopsis thaliana* reads with three pipelines. Bar plot in section 1 (A-Identity, B-length) with coverage cutoff default and in section 2 (A-Identity, B-length) with coverage cutoff 4 for HybPiper BLASTx and DIAMOND.

Additional File 1: Table S1.doc

**Table S1: Table of material with voucher information for all specimens**

Additional File 1 Table S2.doc

**Table S2: Computation time (cumulative) comparison of three different pipelines**

Additional File 1: Table S3.doc

**Table S3: Computation time (cumulative) comparison of three different pipelines in *Arabidopsis thaliana.***

Additional File 1: Table S4.doc

**Table S4. Sample-wise comparison of three pipelines including clean reads, no. of recovered genes, mean recovery of the genes, and standard deviation.**

Additional File 1: Table S5.xls

**Summarized codes of morphological characters with species names. The respective code and its character states are mentioned in the footnotes.**
